# Reporters to mark and eliminate basal or luminal epithelial cells in culture and *in vivo*

**DOI:** 10.1371/journal.pbio.2004049

**Published:** 2018-06-20

**Authors:** Olmo Sonzogni, Jennifer Haynes, Laurie A. Seifried, Yahia M. Kamel, Kai Huang, Michael D. BeGora, Faith Au Yeung, Celine Robert-Tissot, Yujing J. Heng, Xin Yuan, Gerbug M. Wulf, Ken J. Kron, Elvin Wagenblast, Mathieu Lupien, Thomas Kislinger, Gregory J. Hannon, Senthil K. Muthuswamy

**Affiliations:** 1 Cancer Center, Beth Israel Deaconess Medical Center, Harvard Medical School, Boston, Massachusetts, United States of America; 2 Department of Medicine, Beth Israel Deaconess Medical Center, Harvard Medical School, Boston, Massachusetts, United States of America; 3 Princess Margaret Cancer Centre, University Health Network, Department of Medical Biophysics, University of Toronto, Toronto, Ontario, Canada; 4 Campbell Family Institute for Breast Cancer Research, Princess Margaret Cancer Centre, Toronto, Ontario, Canada; 5 Department of Pathology, Beth Israel Deaconess Medical Center, Harvard Medical School, Boston, Massachusetts, United States of America; 6 Cold Spring Harbor Laboratory, Cold Spring Harbor, New York, United States of America; 7 Cancer Research UK Cambridge Institute, University of Cambridge, Cambridge, United Kingdom; Friedrich Miescher Institute for Biomedical Research, University of Basel, Switzerland

## Abstract

The contribution of basal and luminal cells to cancer progression and metastasis is poorly understood. We report generation of reporter systems driven by either keratin-14 (K14) or keratin-8 (K8) promoter that not only express a fluorescent protein but also an inducible suicide gene. Transgenic mice express the reporter genes in the right cell compartments of mammary gland epithelia and respond to treatment with toxins. In addition, we engineered the reporters into 4T1 metastatic mouse tumor cell line and demonstrate that K14+ cells, but not K14− or K8+, are both highly invasive in three-dimensional (3D) culture and metastatic *in vivo*. Treatment of cells in culture, or tumors in mice, with reporter-targeting toxin inhibited both invasive behavior and metastasis *in vivo*. RNA sequencing (RNA-seq), secretome, and epigenome analysis of K14+ and K14− cells led to the identification of *amphoterin-induced protein 2* (*Amigo2*) as a new cell invasion driver whose expression correlated with decreased relapse-free survival in patients with TP53 wild-type (WT) breast cancer.

## Author summary

Most, if not all, cancer-related deaths result from metastasis. The differentiation states of the cancer epithelial cells are thought to be a critical determinant of metastasis. Epithelial cancer cells with a basal cell type are more aggressive in forming metastasis than cancer cells with luminal cell type. Very little is known about how the differentiation states impact metastasis or the molecular mechanisms involved. In this study, we develop and characterize new reporters that fluorescently mark cells in luminal or basal status. These reporters are also coupled to “suicide genes,” which can be used to inducibly and selectively eliminate cells expressing the reporters. We find that elimination of the basal cell type dramatically decreases metastasis and identify amphoterin-induced protein 2 (Amigo2) as a new regulator of cell invasion in basal cells.

## Introduction

Carcinomas are defined as cancers of epithelial cell origin. All carcinomas contain cancer cells in multiple differentiation statuses such as luminal and basal. Keratins (cytokeratins, abbreviated as K) are intermediate filament proteins that are expressed in a differentiation status–specific manner in luminal (K7, K8, K18, K19) or basal (K5, K6, K14, K17) epithelial cells and are routinely used as diagnostic markers for cancer tissues [1,2]. It is thought that cancer epithelia are plastic to an extent and can interconvert between basal and luminal differentiation states during initiation, progression of cancer, and in response to treatment [3]. Therefore, there is a significant need for experimental model systems that facilitate the study of this plasticity and assess the importance of the epithelial differentiation state in modulating the biology of cancer cells.

Despite advances in treatment, patients with breast cancer often relapse and develop metastatic disease, which accounts for over 90% of the 450,000 breast cancer–related deaths each year worldwide [4,5]. Tumor metastasis is a poorly understood, complex, multistep process by which tumor cells disseminate from primary tumors to form secondary tumors [6]. Understanding the key molecular determinants that regulate the metastatic process is fundamental to identifying ways to limit the spread of and to target metastatic breast cancer.

Majority of breast carcinomas express luminal keratins, and a subset express basal keratins [7–9]. Basal keratin expression correlates with higher tumor grade, poor prognosis, and reduced relapse-free and overall survival [7,9,10]. Among the breast cancer subtypes, basal-like (BL) breast carcinomas express both basal and luminal keratins and are associated with aggressive clinical behavior and increased frequency of metastasis compared to luminal subtypes [11]. Recent observations show that collective invasion needs K14-positive leading cells for efficient dissemination and metastases [12]. Conversions of luminal to basal lineage have been observed *ex vivo* in mouse breast cancer models [12], suggesting that lineage plasticity of cancer cells may be involved in the metastatic process. However, the relative contribution of basal versus luminal breast cancer epithelia for tumor progression and metastasis remains unknown.

The goal of the present study is to develop and characterize reporter systems that are more versatile than the frequently used causes recombination (CRE)-based systems. Using the same K14 and K8 promoter used in the CRE expression systems [13], we generated K14 and K8 reporters that coexpress fluorescent proteins and toxin receptors in culture and *in vivo*. The reporters not only mark K14 and K8 positive cells but also enable inducible elimination of specific populations using diphtheria toxin (DT) or ganciclovir (GCV). We demonstrated that elimination of K14 reporter–positive cells decreases invasive phenotype *in vitro* and tumor growth and metastatic load *in vivo*, using 4T1 mammary carcinoma cells [14], a syngeneic, orthotopic transplantation model of metastatic breast cancer. Molecular characterization of K14 reporter cell lines led to the identification of *Amigo2* as a novel mediator of invasion, which demonstrates the utility of the reporter system for understanding biology of metastasis and as a discovery tool to identify novel molecular regulators of cancer metastasis.

Our reporter system has significant benefits over the more frequently used CRE recombinase–based systems [13]. Whereas CRE-based systems are effective in tracing the lineage of marked cells, they do not have the capacity to report plasticity between K8 and K14 differentiation states. Using fluorescence proteins with a very short half-life, we report our ability to monitor changes in K8+ or K14+ status. In addition, we report coexpression of K8 and K14 reporters that can not only allow monitoring transitions between differentiation states but also permit elimination of one cell type while sparing the other. Since differentiation state plasticity plays a critical role in development and cancer, having the ability to eliminate cells in a specific differentiation state is of significant importance. Thus, our model provides the ability to track and control plasticity and hence will provide unprecedented opportunity for those interested is studying K8+ and K14+ epithelial lineages during development and disease.

## Results

### Generation and characterization of targetable K14 reporter and association with invasive behavior in cell culture

**Fig 1:**
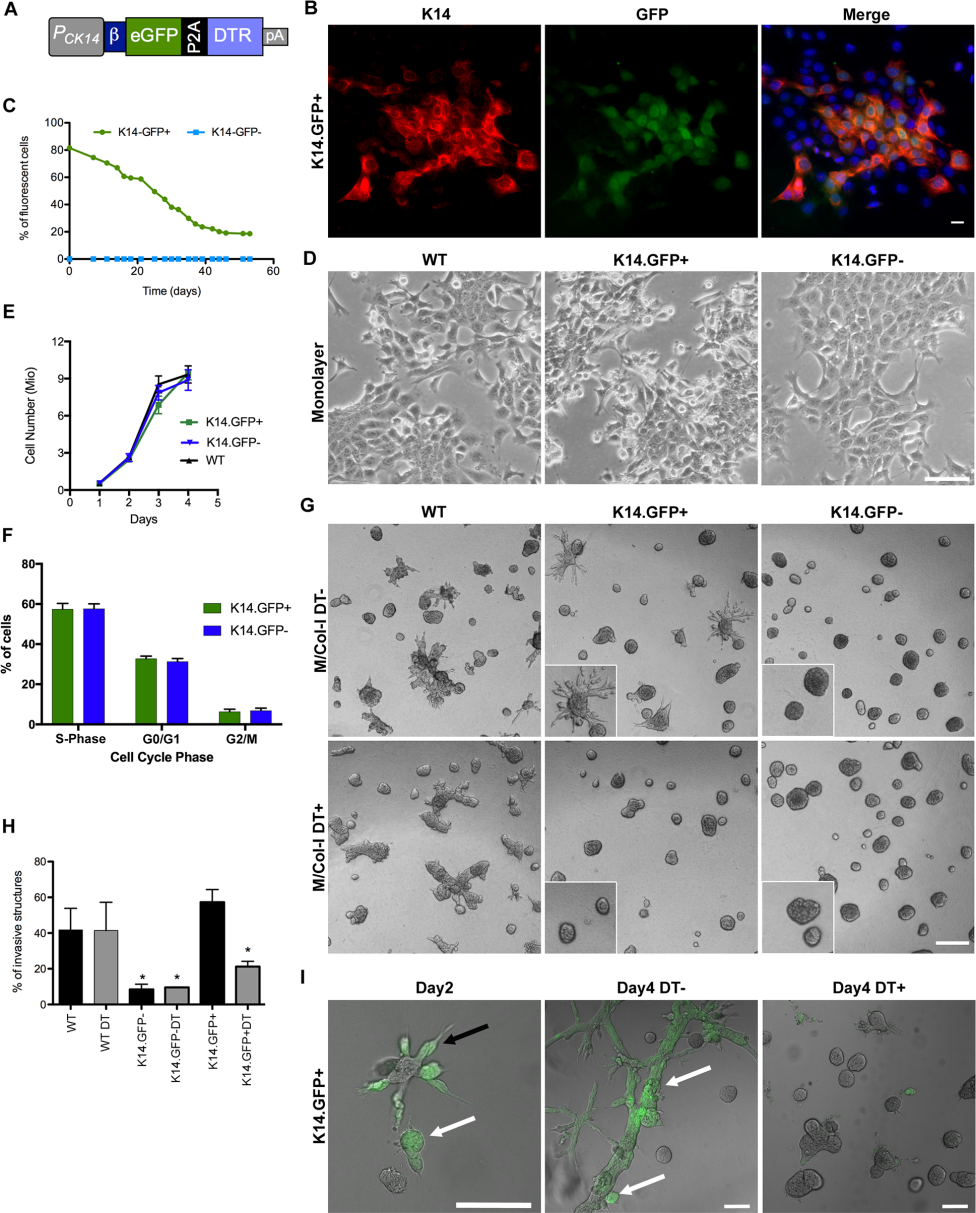
Characterization of K14.GFP reporter and relationship between K14+ and K14− status and invasive behavior of cells in culture. (A) Cartoon of the K14 promoter–driven EGFP-P2A-DTR (K14.GFP) reporter construct. (B) IF shows colocalization of endogenous K14 and GFP in K14.GFP+ monolayer, scale bar 20 μm. (C) Stably transfected K14.GFP reporter cells were sorted by FACS and monitored for changes in percentage of cells expressing GFP by flow cytometry for 54 days. (D) Phase contrast images of K14.GFP+, and K14.GFP− and WT cells grown in 2D, scale bar 100 μm. (E) Changes in cell number over time. Graph shows the mean ± SEM of 3 independent experiments. (F) EdU labeling and flow cytometry to identify cells in different stages of cell cycle. Shown is the mean ± SD of triplicates. (G) K14.GFP+, and K14.GFP− and WT cells grown in 3D (scale bar 200 μm) on top of M/Col-I for 4 days. Two days after seeding, cells were treated with DT (5 ng/ml) for 48 hours (lower panels, DT+). (H) Quantification of invasive structures in G, the data shown are means ± SD from independent experiments carried out in triplicates, at least 300 structures/condition were counted; *p < 0.05 by unpaired *t* test. (I) Phase contrast and GFP overlay images of K14.GFP+ cells grown in 3D in M/Col-I at day 2 and day 4. Black arrow indicates GFP+ cells at the invasive protrusions, white arrows indicate GFP+ in noninvasive 3D structures. DT+ indicates 48-hour exposure to DT, scale bars 100 μm. 3D, three-dimensional; DT, diphtheria toxin; DTR, diphtheria toxin receptor; EdU, 5-Ethynyl-2´-deoxyuridine; EGFP, enhanced green fluorescent protein; FACS, fluorescence-activated cell sorting; GFP, green fluorescent protein; IF, immunofluorescence; K, cytokeratin; M/Col-I, 1:1 mixture of Matrigel/Collagen-I; pA, polyadenylation signal sequence; WT, wild type

Using a human K14 gene promoter developed by the Fuchs laboratory that has been previously shown to drive expression of transgenes in an analogous manner to the endogenous K14 promoter [15–18], we generated a construct that expresses both a fluorescent reporter (enhanced green fluorescent protein [EGFP]) and a monkey diphtheria toxin receptor (DTR) separated by a self-cleaving P2A peptide (Fig 1A). DTR receptor confers sensitivity to DT and has been successfully used for targeted cell ablation in transgenic mice [19–21]. Presence of P2A peptide facilitates translation-coupled cleavage of green fluorescent protein (GFP) and DTR to generate active GFP and DTR products in every cell with active K14 reporter.

The 4T1 mouse mammary carcinoma cell line is comprised of both basal and luminal epithelial cells and forms aggressive BL tumors that metastasize to multiple organs when orthotopically injected into the mammary fat pad of syngeneic BALB/c mice [14]. The K14.GFP reporter was cotransfected with a selection marker containing plasmid, and antibiotic-resistant cell populations were used to enrich for GFP-positive cells by fluorescence-activated cell sorting (FACS). GFP expression was colocalized to endogenous K14, as determined by immunofluorescence (Fig 1B, S1A Fig). Over time, the FACS-sorted K14.GFP+ cell population gave rise to a GFP-negative (K14.GFP−) population, suggesting that the K14-positive lineage was not terminally differentiated. In contrast, the K14.GFP− cell population did not give rise to a K14.GFP+ population, even when cultured for extended periods (>50 days), suggesting a hierarchical relationship between K14-positive and K14-negative states under normal cell culture conditions (Fig 1C). The K14.GFP− population, generated from the K14.GFP+ population, retained the reporter plasmid in the genomic DNA (S1B Fig), demonstrating that the K14.GFP promoter was present but not active in K14.GFP− cells.

In monolayer cultures, K14.GFP− cells formed the characteristic cobblestone epithelial morphology, with clearly identifiable cell–cell contacts, whereas the K14.GFP+ cell population was pleomorphic (Fig 1D). The presence of rounded cells in the K14.GFP+ population prompted us to investigate whether K14.GFP+ and K14.GFP− cells differ in their proliferation rates. Surprisingly, cell proliferation measured using 3-(4,5-dimethylthiazol-2-yl)-2,5-diphenyltetrazolium bromide (MTT) assay or changes in total cell number or 5-ethynyl-2′-deoxyuridine (EdU) incorporation combined with DNA staining for cell cycle analysis did not show any difference between K14.GFP+ and K14.GFP− cell populations. (Fig 1E and 1F, S1C and S1D Fig). K14-positive cancer cells are associated with aggressive behavior in culture and *in vivo* [12]. Consistent with this logic, about 50% of K14.GFP+ and wild-type (WT) cells formed invasive structures, bearing multiple invasive projections when plated in a 3D culture on a bed of a 1:1 mixture of Matrigel/Collagen-I (M/Col-I). In contrast, less than 10% of K14.GFP− cells formed invasive structures (Fig 1G and 1H). To determine if the expression of DTR would provide us the ability to eliminate K14.GFP+, we treated cells with DT. DT addition to the K14.GFP+ population resulted in an almost complete loss of structures with invasive protrusions (Fig 1G and 1H), whereas DT did not affect WT or K14.GFP− cells, demonstrating the utility of DT treatment to eliminate K14+ cells with invasive properties.

Previous studies have shown that K14+ cells are present at the leading edge of invasive protrusions [12]. To understand the relationship between our K14.GFP reporter expression and cell invasion, we monitored GFP expression in 3D culture. We observed that GFP-expressing cells were present at both invasive protrusions and in noninvasive 3D structures, demonstrating that our K14.GFP reporter marks cells irrespective of the invasion behavior (Fig 1I). We also noted that not all GFP-expressing cells were lost upon DT treatment in 3D (Fig 1I) and in monolayer cultures (S1E Fig).

### K14.GFP+ cells promote increased metastatic burden *in vivo*

**Fig 2:**
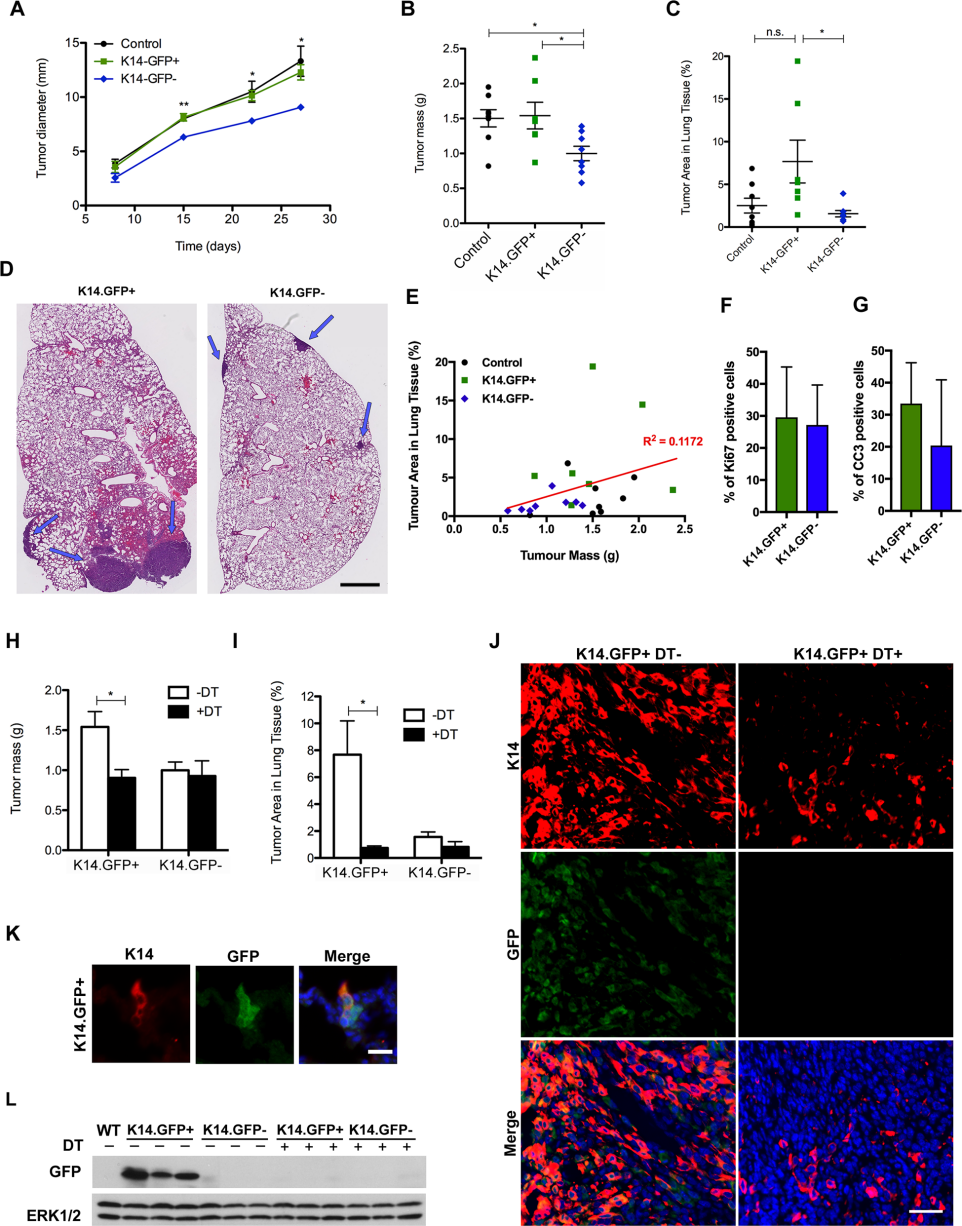
K14.GFP+ cells have greater metastatic potential that K14.GFP− cells *in vivo*. (A) Primary tumor diameters from mice injected with 4T1 control, or K14.GFP reporter cell lines (K14.GFP+ or K14.GFP−), measured over the course of the experiment. *n* = 8 (control), 7 (K14.GFP+), and 8 (K14.GFP−); *p < 0.05, **p < 0.001 by one-way ANOVA followed by Newman-Keuls multiple comparisons posttest. (B) Final tumor masses measured after mice were euthanized. (C) Lung metastases were quantified by measuring the average percent area of lung tissue occupied by the tumor in 5 sections (5-μm thick) cut at 200-μm intervals. (D) Representative images of H&E-stained lung sections containing metastases from mice injected with K14.GFP+ or K14.GFP− cell lines. Arrows indicate metastasis; scale bar 1 mm. (E) Correlation analysis of primary tumor mass and lung metastasis. Scatter plot of the percent tumor area in lung tissue compared to the primary tumor mass for each mouse analyzed (*n* = 23). The slope does not significantly differ from zero by linear regression analysis (*p* = 0.1097). Quantification of percent of positive cells for Ki67 (F) and CC3 (G) in tumors from K14.GFP+ and K14.GFP− cells. Shown are means ± SD of quantifications of whole tumor sections. (H) Final tumor masses measured after mice were euthanized. For DT treatment, the mice were injected i.p. with DT (25 mg/kg) on days 7, 9, 11, and 13. *n* = 7 (K14.GFP+; no DT), 4 (K14.GFP+; with DT), 8 (K14.GFP−; no DT), and 4 (K14.GFP−; with DT). *p* = 0.0425 by unpaired *t* test. (I) Lung metastases were quantified for the mice described in H. Statistical analysis for (B), (C), and (I) was calculated by one-way ANOVA followed by Tukey’s multiple comparisons posttest; *p < 0.05 or n.s. (J) Fluorescent IHC was performed for K14 and GFP on primary tumors generated from K14.GFP+ cell lines either DT− or DT treated (“DT+”) as described in (H); scale bar 40 μm. (K) Same staining as described in J was carried out on metastatic lung of K14.GFP+-injected mice; scale bar 20 μm. (L) Immunoblots of lysates from primary tumors were analyzed for GFP expression. Every lane represents a different tumor. Blots were also probed with antibodies for ERK1/2 as loading control. If not otherwise indicated, all graphs show mean ± SEM. DT, diphtheria toxin; ERK1/2, extracellular signal-regulated kinase 1/2; GFP, green fluorescent protein; H&E, hematoxylin and eosin; IHC, immunohistochemistry; i.p., intraperitoneally; K, cytokeratin; n.s., not significant

The results obtained with the 4T1 model *in vitro* prompted us to investigate the *in vivo* behavior of the K14 reporter cell lines. Control, K14.GFP+, or K14.GFP− cells were injected orthotopically into the abdominal mammary fat pad of syngeneic BALB/c mice. While all mice receiving injections developed fast-growing tumors, primary tumors derived from K14.GFP− cells grew slower than control or K14.GFP+ cells (Fig 2A).

All mice were euthanized 4 weeks after injections when the largest tumor reached 1.5 cm in diameter. Tumors derived from control or K14.GFP+ cells had a significantly larger mass compared to those generated by K14.GFP− cells (Fig 2B). Histological examination of tumors showed that both K14.GFP+ or K14.GFP− cells were similar with regard to the cellular morphology, tissue organization, and extent of necrosis (S2A Fig). However, examination of lung sections revealed that metastatic burden was significantly increased in mice injected with K14.GFP+ cells compared to those injected with K14.GFP− cells, as quantified by percentage of total lung area occupied by tumors (Fig 2C and 2D). Lung metastasis was also enhanced for K14.GFP+ cells relative to that of control cells; however, the difference was not statistically significant (Fig 2C). Although both tumor size and lung metastasis were greater for the K14.GFP+ group, correlation coefficient analysis of matched pairs of primary tumors and lung metastases from all groups showed that lung metastasis did not correlate with primary tumor mass (Fig 2E), demonstrating that the increase in lung metastasis is not simply due to the increase in mass of the primary tumor in mice injected with K14.GFP+ cells. Thus, K14.GFP+ cell populations form more aggressive tumors *in vivo* with increased metastatic potential compared to K14.GFP− cells.

Next, we investigated cell proliferation and cell death in primary tumors to understand why K14.GFP+ cells form faster-growing primary tumors. Surprisingly, neither Ki67 (a proliferation marker) nor cleaved caspase-3 (an apoptosis marker) was significantly different between K14.GFP+ and K14.GFP− tumors (Fig 2F and 2G and S2B Fig). Since increase in tumor size can be due to increased vascularization, we also evaluated the expression of the endothelial marker CD31 in the K14.GFP+ and K14.GFP− tumors, but no significant difference was found (S2C Fig). These results are consistent with the lack of a difference in cell proliferation rates between K14.GFP+ and K14.GFP− cell lines in culture. It is likely that differences in the size of tumors derived from K14.GFP+ or K14.GFP− are due to cell intrinsic or extrinsic factors, which remain to be understood (see Discussion).

### DTR-mediated ablation of K14.GFP+ cells *in vivo* decreases primary tumor mass and lung metastases

To assess the importance of the K14.GFP+ population for primary tumor growth and metastasis formation, we investigated the effects of eliminating K14.GFP+ cell population *in vivo*. Mice with approximately 3.0-mm tumors generated using K14.GFP+ or K14.GFP− cells were injected with DT every other day for 7 days. The mice were euthanized 4 weeks after cell injections and evaluated for primary tumor and lung metastases. DT administration significantly reduced both primary tumor mass and lung metastasis in mice injected with K14.GFP+ cells but had no impact on the K14.GFP− group (Fig 2H and 2I). These results support the notion that K14+ cells are critical regulators of metastatic spread and demonstrate the utility of the reporter to investigate the role of K14+ populations *in vivo*.

### Characterization of K14.GFP in tumors and metastasis

As expected, tumors generated from K14.GFP+ cells had high frequency of K14-positive cells compared to tumors generated from K14.GFP− cells, as determined by immunofluorescence. Coimmunostaining with anti-GFP antibodies showed colocalization of GFP with K14 immunostaining in tumors generated from K14.GFP+ cells but not in tumors generated from K14.GFP− cells or DT treated (Fig 2J and S3A Fig). Furthermore, K14 and GFP colocalized in the metastatic lesions present in the lung of mice with K14.GFP+ tumors but not in K14.GFP− tumors or DT treated (Fig 2K and S3B Fig). Together, these observations demonstrate that expression of the reporter was associated with endogenous K14 expression in both primary tumor and metastasis.

Consistent with the observations in tumor and lung sections obtained from K14.GFP− or DT-treated mice, anti-GFP immunoblot analysis did not show expression of GFP in those tumors (Fig 2L), demonstrating that DT administration was effective in eliminating reporter-expressing cells.

### K14 and K8 reporters for labeling and selective elimination of cell populations

**Fig 3:**
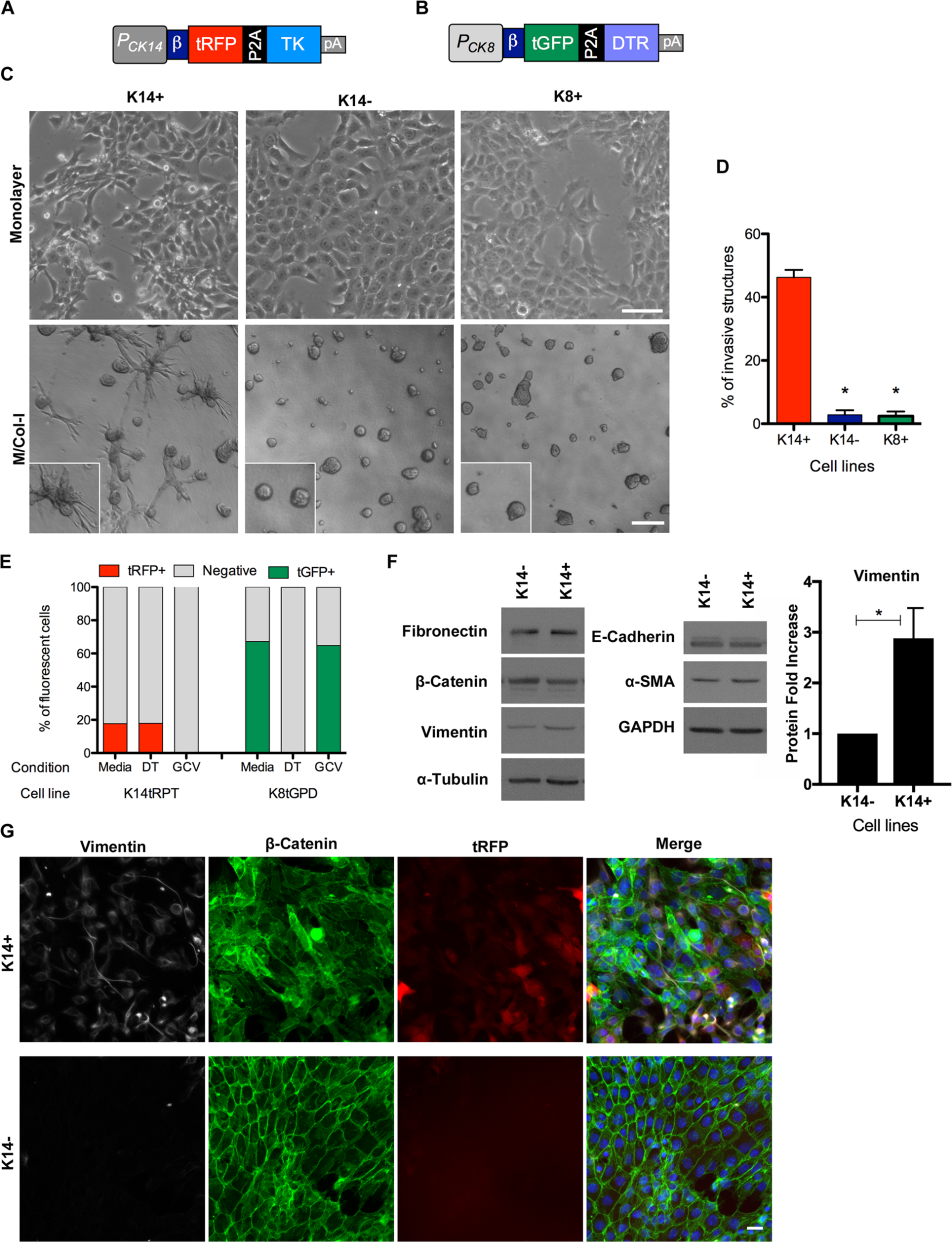
Generation and characterization of 4T1 cell lines expressing K14 and K8 reporters. (A) Cartoon of the K14 promoter–driving expression of tRFP-P2A-TK (K14.tRPT) reporter. (B) Cartoon of the K8 promoter–driving expression of tGFP-P2A-DTR (K8.tGPD) reporter construct. (C) Phase contrast images of K14+, K14−, and K8+ cells grown in monolayer (upper panels, scale bar 100 μm) and 3D (lower panels, scale bar 200 μm) on M/Col-I culture for 4 days. (D) Quantification of invasive structures in C, represented as means ± SEM from 3 independent experiments; *p < 0.001 by unpaired *t* test. (E) K14+ and K8+ cells were treated with DT (2.5 ng/ml), GCV (1 μg/ml), or media. Column bars indicate the percentage of reporter-positive and negative cells after treatments for the indicated cell lines, as determined by flow cytometry. (F) Immunoblots of lysates from K14+ and K14− reporter cell lines were analyzed for changes in expression of E-cadherin, β-catenin, fibronectin, vimentin, and α-SMA. Blots were also probed with antibodies for GAPDH and α-tubulin as loading controls. Right panel shows quantification of vimentin for 3 independent western blot *p* = 0.0317 by paired *t* test; mean ± SD is shown. (G) Immunofluorescence of K14+ (upper panel) and K14− (lower panel) monolayers for vimentin, β-catenin, or detection of endogenous tRFP signal. Scale bar 25 μm. α-smooth muscle actin; DT, diphtheria toxin; DTR, diphtheria toxin receptor; GAPDH, glyceraldehyde 3-phosphate dehydrogenase; GCV, ganciclovir; K, cytokeratin; K8.tGPD, keratin-8 promoter followed by turbo green fluorescent protein and diphtheria toxin receptor; K14.tRPT, keratin-14 promoter followed by a turbo red fluorescent protein and herpes simplex virus thymidine kinase; M/Col-I; 1:1 mixture of Matrigel/Collagen-I; pA, polyadenylation signal sequence; TK, thymidine kinase; tGFP, turbo green fluorescent protein; tRFP, turbo red fluorescent protein

During DT-mediated depletion of K14.GFP+ cells in monolayer cultures, 1% to 2% of GFP-positive cells remained, even after prolonged DT treatment (S1E Fig). We reasoned that this may be because of the long half-life (approximately 26 hours) of the GFP protein [22]. To rule out the effect of protein half-life on the presence of GFP-positive cells, we generated a new set of reporters carrying a fast-maturating and short-half-life (2.0 hours) fluorescent protein coupled with a suicide gene. In addition to K14, we also generated a reporter to mark K8-positive cells. The fluorescent protein and toxin were chosen such that they can be coexpressed in the same cell to better monitor changes in the cell differentiation state. K14 promoter followed by a turbo red fluorescent protein (tRFP) and the suicide gene herpes simplex virus thymidine kinase [23] (TK; K14.tRPT) and a K8 promoter followed by turbo green fluorescent protein (tGFP) and DTR (K8.tGPD) were stably transfected into 4T1 (Fig 3A and 3B).

To directly compare K14+ and K8+ cells, we established populations of K14.tRFP- and K8.tGFP-positive cells by performing 2 sequential rounds of FACS sorting. Similar to the K14.GFP+, the K14.tRPT+ (referred to as K14+) population gave rise to K14.tRPT− (referred to as K14−) cells over time, but the K14− cells did not generate K14+ cells. In contrast, K8+ cells did not show any significant decrease in the percentage of reporter-positive cells over multiple passages (S4A Fig). Endogenous K8 and K14 in the K8+, K14+, and K14− monolayers colocalized with tGFP and K14 expression with tRFP expression (S4B and S4C Fig, respectively).

As expected, K14+ cell populations had a less organized monolayer as compared with K14− or K8+ populations (Fig 3C). As observed for K14.GFP+ cells, almost 50% of K14+ cells formed highly invasive structures of variable size in M/Col-I basement membrane matrix, whereas the K8+ and K14− population showed almost exclusively noninvasive structures (Fig 3C and 3D). Consistently, K14+ were found to migrate faster than K14− in a scratch assay (S4D Fig). In addition, the K14+ and K14− cells did not show any detectable difference in their proliferation rates, as determined by MTT and EdU assays (S5A and S5B Fig).

To demonstrate the utility of the inducible killing strategy, we exposed monolayers to GCV for K14 reporter–expressing TK and to DT for K8 reporter–expressing DTR. Toxin treatment led to complete ablation of reporter-positive cells (Fig 3E and S5C Fig), confirming that the system would be a reliable method to deplete reporter-positive cells.

### K14 reporter–expressing cells have features associated with mesenchymal transition

To determine if the invasive behavior in culture and the metastatic properties *in vivo* are associated with mesenchymal characteristics in K14 positive cells, we compared K14+ and K14− cells for changes in expression of epithelial and mesenchymal markers. Among the mesenchymal markers analyzed, we observed a 3.0-fold increase in the levels of vimentin (Fig 3F). Immunofluorescence analysis showed an increase in vimentin expression in the majority of the K14+ cells, demonstrating that increase in vimentin protein levels is not restricted to a subpopulation of K4+ cells (Fig 3G). Interestingly, although β-catenin protein levels did not change, immunofluorescence analysis demonstrated a loss of cell–cell junction localization in K14+ cells and a gain in cytoplasmic and nuclear signal (Fig 3G). Similar changes in vimentin expression and β-catenin localization were observed in K14.GFP+ cell monolayers and tumors (S6A and S6B Fig). Consistent with β-catenin mislocalization from cell–cell junctions, E-cadherin localization was altered in K14.GFP+ cells in culture and in tumors *in vivo* (S7A and S7B Fig). These observations demonstrate that K14+ have mesenchymal plasticity through which they coexpress both mesenchymal and epithelial markers.

Because epithelial–mesenchymal transition (EMT) has been linked with increased cancer stem cell properties [24], we reasoned that it could be the reason why K14.GFP+ tumors are bigger than K14.GFP−. To evaluate the percentage of cells that would potentially have cancer stem cell features, we analyzed the surface expression of CD44 and CD24 in both our K14-derived cell lines but did not find any differences between K14+, K14−, K14.GFP+, and K14.GFP− cells (S7C and S7D Fig).

### Generation and basic characterization of transgenic mice carrying K14 and K8 reporter

**Fig 4:**
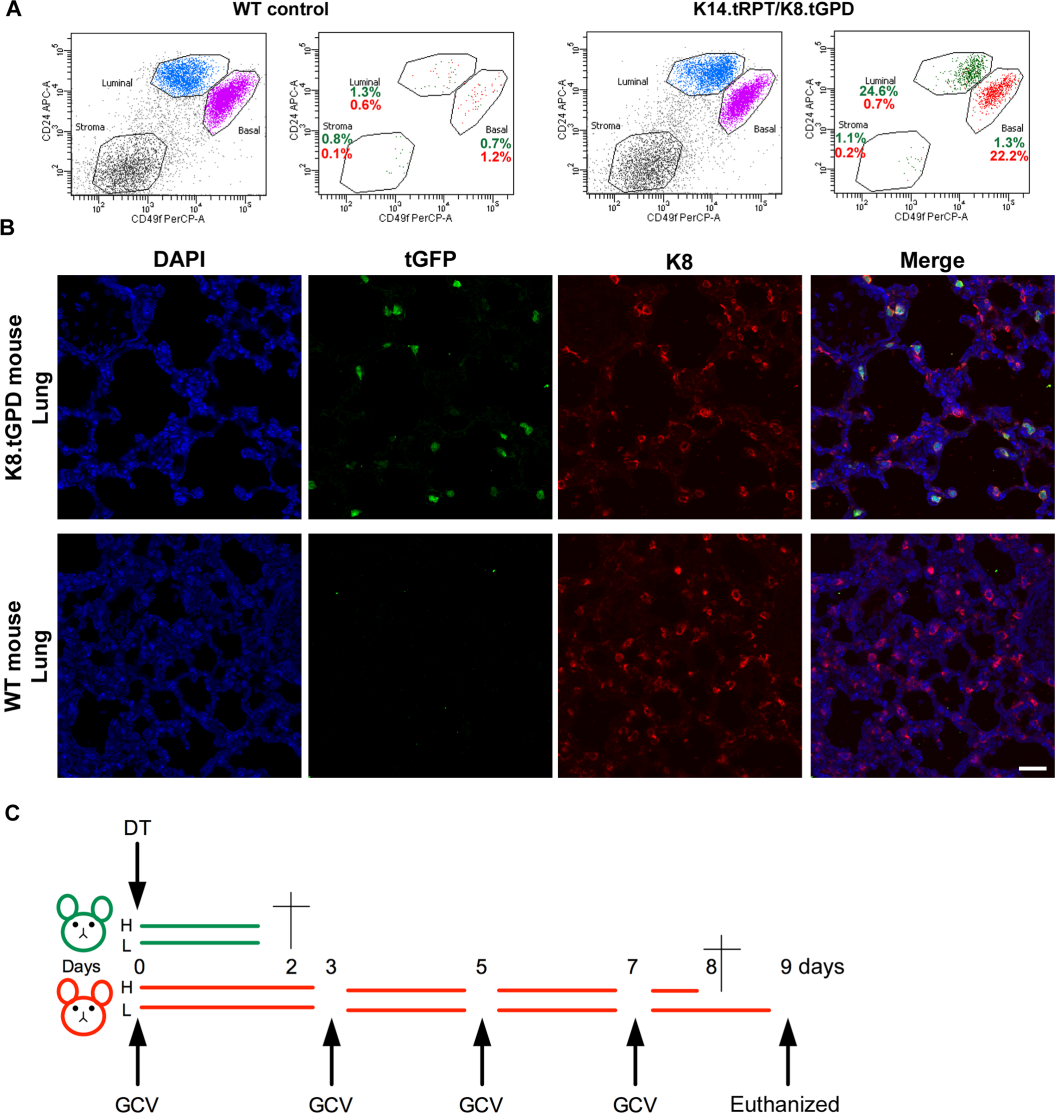
Generation and characterization of transgenic mice expressing K14 and K8 reporters. (A) Flow cytometry analysis for stromal, basal, and luminal compartments of cells from mammary glands of control or K14.tRPT/K8.tGPD double-positive transgenic mouse. For each type of mouse, the first dot plot shows the total population in each cell compartment, whereas the second plot shows only the cells that are positive for the reporters. Gates were set based on the negative control and dots pseudocolored to represent the reporter-positive cells. (B) Fluorescent IHC showing colocalization of tGFP with endogenous K8 in the lung of a K8.tGPD-positive mouse (upper panels). Lower panel shows the control staining on a WT mouse in which no tGFP was detected; scale bar 10 μm. (C) Positive and negative mice were injected i.p. with either high (“H”; GCV = 100 μg/g; DT = 50 ng/g) or low (“L”; GCV = 20 μg/g; DT = 10 ng/g) doses at indicated time points (days). DAPI, 4’,6-diamidino-2-phenylindole; DT, diphtheria toxin; GCV, ganciclovir; IHC, immunohistochemistry; i.p., intraperitoneally; K8.tGPD, keratin-8 promoter followed by turbo green fluorescent protein and diphtheria toxin receptor; K14.tRPT, keratin-14 promoter followed by a turbo red fluorescent protein and herpes simplex virus thymidine kinase; tGFP, turbo green fluorescent protein; WT, wild-type

To expand the utility of our reporters for *in vivo* studies, we generated 2 transgenic Friend leukemia virus B (FVB) mice expressing either the K8.tGPD or the K14.tRPT construct (Fig 3A and 3B). Male mice carrying the K14.tRPT reporter were nearly sterile, likely because of the toxicity associated with the TK gene [23], but females showed normal fertility. To determine the ability of reporters to mark appropriate cell compartment *in vivo*, we analyzed the relationship between expression of reporter gene and epithelial differentiation status in the mammary gland. Primary mouse mammary epithelial cells were analyzed using well-characterized luminal and basal cell surface markers [25] in conjunction with expression of reporter genes. The K8.tGFP reporter–expressing cells were restricted to the luminal compartment, and the K14.tRFP reporter–expressing cells were restricted to the basal (Fig 4A and S8A Fig) compartment, demonstrating the ability of the reporters to mark appropriate cell populations *in vivo*.

Percentage of reporter-positive cells showed modest variation among mice, but the fluorescent protein was always restricted to the right compartment of mammary gland epithelia (S8B Fig). In addition, immunohistochemistry analysis of the lung shows tGFP expression specifically restricted to K8-positive cells (Fig 4B), demonstrating the specificity of the reporters in organs other than the mammary gland. To assess if the suicide genes were effective *in vivo*, we injected reporter-positive and WT animals with either a high or a low dose of GCV or DT intraperitoneally (i.p.). DT administration induced a lethal response within 48 hours for the K8.tGPD line, (Fig 4C) likely due to elimination of differentiated epithelial cells in most of the internal organs. In K14.tRPT mice, both high and low doses of GCV caused massive weight loss or a lethal response. Nontransgenic mice did not show any phenotype in response to DT or GCV administration. Hematoxylin and eosin (H&E) analysis of the liver of DT-treated K8.tGPD mouse showed a decreased number of cells compared to DT-treated WT mouse (S8C Fig). For K14.tRPT-positive but not WT mice, GCV-treated mammary gland showed a loss of the mammary ductal organization and an increased lymphocyte infiltration, likely due to inflammation induced by GCV-induced cell death and a significant change in adipose tissue where the normal fat tissues were replaced by brown fat–like tissues, presumably due to the large consumption of energy to compensate for cell loss, which turns energy-storing mature fat cells into energy-burning brown fat cells. (S8D Fig).

These results demonstrate that the transgenic mice express the reporter in the right compartments and that the suicide genes that respond to the stimuli making cells from these mouse models can be used in orthotopic transplantation or *in vitro* differentiation settings as powerful tools for the investigation of epithelial lineage biology in culture and *in vivo*.

### Molecular characterization of differences between 4T1 K14+ and K14− cell lines

To gain insight into the molecular basis for the phenotypic differences in invasive, tumorigenic, and metastatic behaviors observed between K14+ and K14− cells, we conducted secretome, RNA sequencing (RNA-seq), and epigenome analysis.

**Fig 5:**
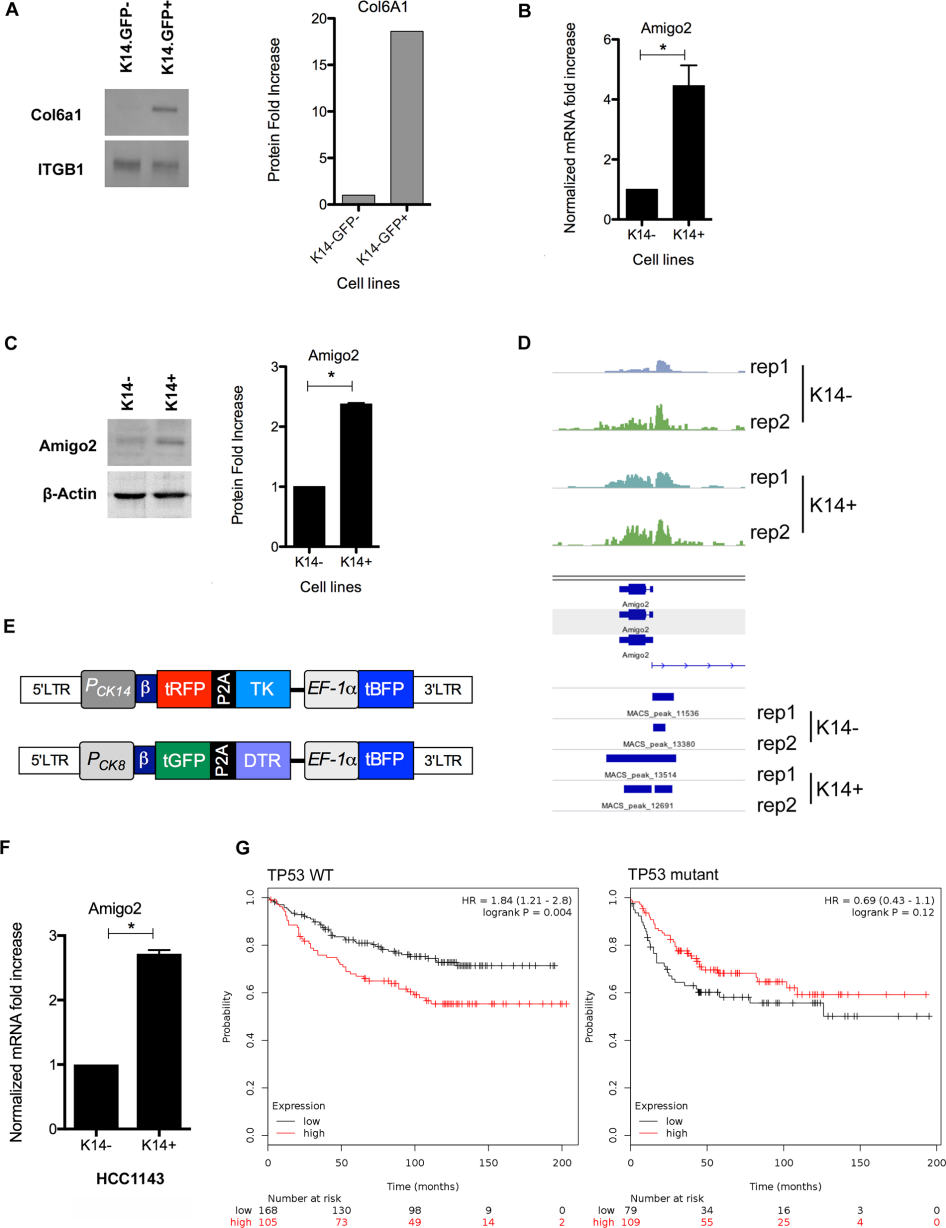
K14+ cells secrete more Col6a1 and express higher levels of Amigo2 than K14− cells. (A) Anti-Col6a1 immunoblots of secreted proteins from K14.GFP+ or K14.GFP− reporter cell lines. CM were concentrated and analyzed for levels of secreted Col6a1. ITGB1, also present in the secretome, was used for loading control. (B) RT-PCR for *Amigo2* mRNA level on K14+ and K14− cells. Results show the mean ± SD of 3 independent experiments, *p* = 0.0126 by paired *t* test. (C) Amigo2 protein level was detected in K14+ and K14− cell lysates by western blot. Quantification of 3 independent experiments, *p* < 0.0001 by paired *t* test; mean ± SD is shown. (D) Chip for H3K27Ac shows the magnitude of the peaks for K14+ and K14− replicates at the *Amigo2* locus. (E) Cartoon of self-inactivating lentiviral K14.tRPT (upper cartoon) and K8.tGPD reporters (lower cartoon). (F) RT-PCR analysis of *Amigo2* mRNA expression in K14+ or K14− human breast cancer cell line HCC1143. Quantification of independent experiments in triplicates *p* = 0.0148 by paired *t* test; mean ± SD is shown. (G) Kaplan-Meier plot in TP53 mutant and TP53 WT breast cancer show relationship between *Amigo2* expression and relapse-free survival. Amigo2, amphoterin-induced protein 2; Chip, chromatin immunoprecipitation; CM, conditioned medium; Col6a1, Collagen VI subunit A; DTR, diphtheria toxin receptor; *EF-1α*, *elongation factor 1α*; GFP, green fluorescent protein; H3K27Ac, histone 3 lysine 27; ITGB1, integrin β-1; K, cytokeratin; K8.tGPD, keratin-8 promoter followed by turbo green fluorescent protein and diphtheria toxin receptor; K14.tRPT, keratin-14 promoter followed by a turbo red fluorescent protein and herpes simplex virus thymidine kinase; LTR, long terminal repeat; RT-PCR, real-time PCR; tBFP, turbo blue fluorescent protein; tGFP, turbo green fluorescent protein; TK, thymidine kinase; tRFP, turbo red fluorescent protein; WT, wild-type.

Secreted proteins can modify the extracellular matrix and are therefore potential key candidates to influence invasion and metastasis. Mass spectrometry analysis of conditioned media (CM) from WT, K14.GFP+, and K14.GFP− cells identified approximately 1,900 proteins (S1 Data). The Collagen VI subunit A (*Col6a1*) was the most differentially secreted protein between K14.GFP+ and K14.GFP− cells (Table 1), with an approximately 10-fold increase in abundance for K14.GFP+ compared to K14.GFP− cells. Immunoblot analysis of CM confirmed that *Col6a1* was highly secreted by K14.GFP+ cells and relatively undetectable in K14.GFP− cells’ CM (Fig 5A). Two recent secretome studies reported that secreted *Col6a1* levels are increased for several strongly metastatic cancer cell lines relative to their less-metastatic counterparts [26,27]. *Serpine2* was also found to be more secreted by K14+ cells, and its expression and secretion have been correlated with metastatic disease [28]. Other proteins that showed at least 4-fold difference in levels are listed in Table 1. These results provide a strong validation of our reporter system and its potential to uncover hypothesis-generating observations.

**Table 1:** Proteins identified by secretome analysis as most highly secreted for K14+ compared to K14− cells.

Symbol	Protein Name	Fold change
Col6a1	Collagen, type VI, alpha-1	9.94
Olfml2b	Olfactomedin-like protein 2B	7.53
Serpine2	Serine peptidase inhibitor, clade E, member 2	6.73
Col7a1	Collagen, type VII, alpha-1	6.29
Sf3a3	Splicing factor 3A subunit 3	4.79
Hspa4l	Heat shock 70 kDa protein 4L	4.68

Includes proteins that had an average count of 3 within the replicates and at least a 4-fold difference between cell lines.

To identify genes that are differentially expressed, we performed RNA-seq analysis for WT, K14.GFP+, and K14.GFP− cells (S2 Data). Interestingly, among the transcripts most differentially expressed between K14.GFP+ and K14.GFP− cells, we found both new and known metastasis-associated genes (Table 2). Genes including metallothionein-2 (*Mt2*) [29,30], transmembrane glycoprotein nonmetastatic B **(***Gpnmb*) [31], caveolin 1 (*Cav1*) [32], *Col6A1* [26,27], HLA-DR antigens-associated invariant chain (*Cd74*) [33,34], secretory leukocyte peptidase inhibitor (*SLPI*) [35], carbonic anhydrase (*Car9*), amphiregulin (*Areg*) [36–38], and peripheral myelin protein 22 (*PMP22*) [39] have previously been reported in association with invasive and/or metastatic phenotypes or as markers for poor prognosis in human breast cancer.

**Table 2:** Genes identified by RNA-seq analysis as most highly expressed in K14+ compared to K14− cells.

Symbol	Gene Name	Fold change (log2)
Mt2	Metallothionein 2	3.16
Gpnmb	Glycoprotein (transmembrane) nmb	3.00
Amigo2	Adhesion molecule with Ig like domain 2	2.97
Cav1	Caveolin 1	2.88
Col6a1	Collagen, type VI, alpha-1	2.77
Cd74	Invariant polypeptide of major histocompatibility complex, class II antigen-associated	2.47
Car9	Carbonic anhydrase 9	2.36
Slpi	Secretory leukocyte peptidase inhibitor	2.36
Areg	Amphiregulin	2.36
Pmp22	Peripheral myelin protein 22	2.29
Cyp2c55	Cytochrome P450, family 2, subfamily c, polypeptide 55	2.21

Includes genes that were highly abundant (FPKM > 5) and had at least a log2 difference of 2 between cell lines.

**Abbreviations:** FPKM, fragments per kilobase per million reads; RNA-seq, RNA sequencing

We pursued analysis of a transmembrane protein, amphoterin-induced protein 2 (*Amigo2)*, thought to be involved in regulation of cell–cell interactions [40] but not known to be involved in regulation of cell invasion. We validated the RNA-seq data by demonstrating that mRNA and the protein levels of *Amigo2* were significantly higher in K14+ 4T1 cells as compared to K14− (Fig 5B and 5C). To determine if the difference in *Amigo2* gene expression relates to underlying differences in the epigenetic states in the *Amigo2* gene, we performed chromatin immunoprecipitation sequencing (ChIPseq) analysis for acetylation of histone 3 lysine 27 (H3K27Ac), which is a marker for active chromatin [41]. In two independent analyses, a stronger signal at the *Amigo2* locus was consistently observed in the K14+ cells as compared with the K14− counterpart (Fig 5D and S9A Fig).

### *Amigo2* and breast cancer

To determine if differential expression of *Amigo2* is observed in other models of breast cancer, we used HCC1143, a human breast cancer cell line that is known to express both luminal and basal keratins [42]. To facilitate transduction of human cells, we regenerated K8 and K14 reporter in a self-inactivating (SIN) lentiviral-based expression vector (Fig 5E) that constitutively coexpresses a blue fluorescence protein (tBFP). Cells were transduced and sorted first for tBFP and subsequently for expression of either tRFP (K14) or tGFP (K8) (S9B Fig). Consistent with what we found for 4T1 cells, HCC1143 K14.tRFP+ populations had significantly higher levels of *Amigo2* mRNA compared with the K14− population (Fig 5F), demonstrating the relationship between K14 status and *Amigo2* expression in human breast cancer cells.

To gain insight into the role of *Amigo2* in human breast cancer, we analyzed the relationship between *Amigo2* levels in different breast cancer subtypes. A high level of *Amigo2* expression was not associated with prognosis in all breast cancers, suggesting that it is not an independent prognostic indicator (S10A Fig). However, a high level of *Amigo2* was associated with poor relapse-free survival, irrespective of estrogen receptor (ER) status of the tumors (S10B Fig), but had no predictive value associated with human epidermal growth factor receptor 2 (HER2) status (S10C Fig). Interestingly, high *Amigo2* was significantly associated with poor relapse-free survival in patients with TP53 WT and not in patients with TP53 mutant breast cancers (Fig 5G), [43] identifying an intriguing relationship between TP53 status and *Amigo2* biology in breast cancer.

### *Amigo2* as a regulator of invasive behavior

**Fig 6:**
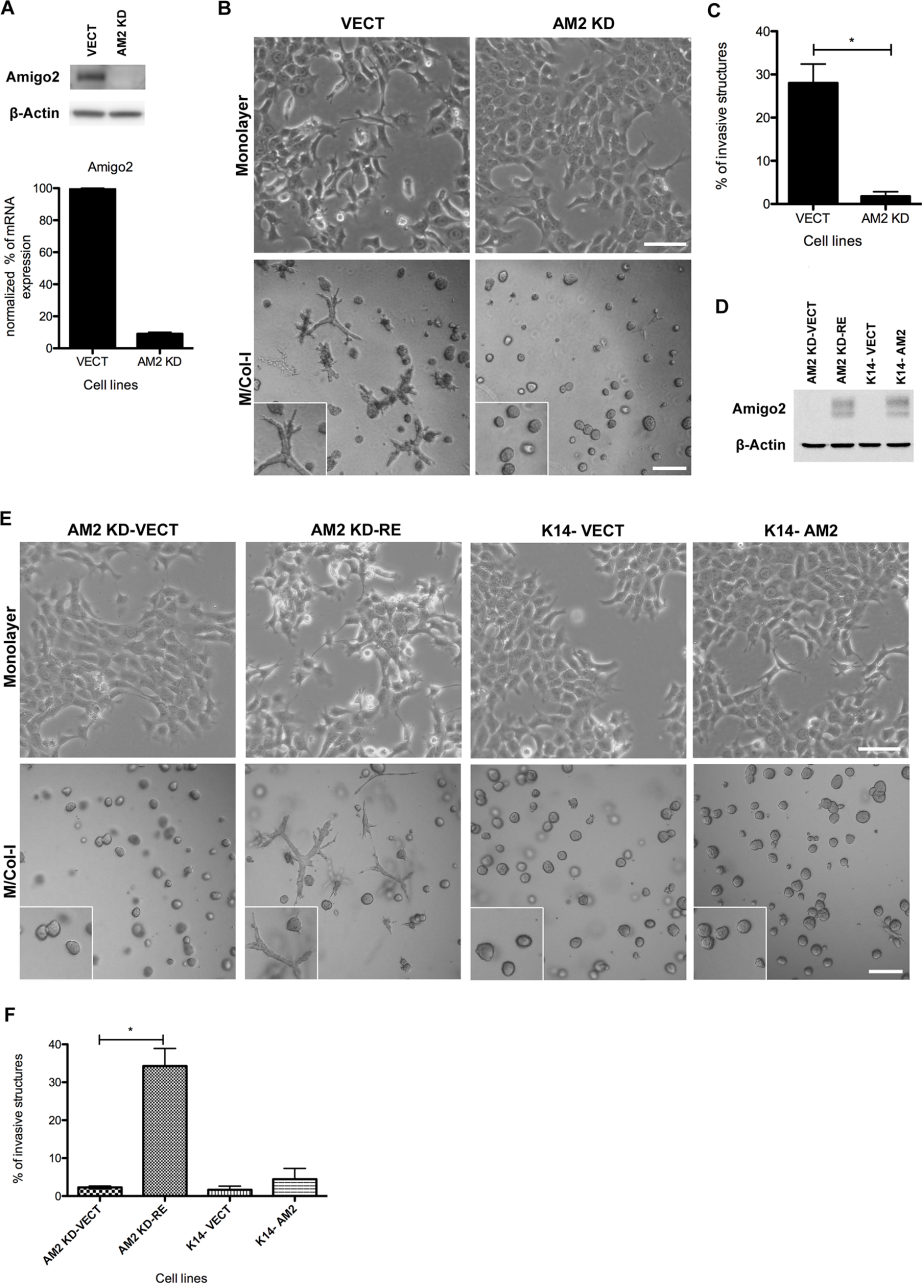
Amigo2 a new regulator of breast cancer cell invasion. (A) Amigo2 protein level (western blot) and mRNA expression (RT-PCR) in control cells (VECT) or AM2 KD. Results were normalized on GAPDH. (B) Images of monolayers (upper panels) or 3D (lower panels) for 4T1 cells VECT or AM2 KD. (C) Invasive structures from 3 independent experiments were quantified; mean ± SEM is shown; *p* = 0.0044 by unpaired *t* test. (D) Western blot analysis of control (AM2 KD-VECT and K14-VECT) or Amigo2 rescue (AM2 KD-RE and K14-AM2) 4T1 cell lines. (E) Images of cells grown in monolayer (upper panels) or in 3D (lower panels). (F) Quantification of invasive structures from 3 independent experiments; mean ± SEM is shown; *p* = 0.0024 by unpaired *t* test. All 3D images of M/Col-I cultures were taken on day 4 postseeding. 2D scale bar 100 μm; 3D scale bar 200 μm. 3D, three-dimensional; AM2 KD, Amigo2 knock-down cells; AM2 KD-RE, *Amigo2* knock-down rescue; AM2 KD-VECT, *Amigo2* knock-down control vector; Amigo2, amphoterin-induced protein 2 GAPDH, glyceraldehyde 3-phosphate dehydrogenase; K14-AM2, cytokeratin 14 negative *Amigo2* overexpressing cell; M/Col-I, 1:1 mixture of Matrigel/Collagen-I; RT-PCR, real-time PCR; VECT, control vector

To determine if *Amigo2* regulates cell invasion, we knocked down its expression in 4T1 cells. *Amigo2* knock-down (AM2 KD) cells have a dramatic decrease in the protein levels of *Amigo2* and a 90% decrease in mRNA expression as compared to the control vector (VECT)-transduced cells (Fig 6A). AM2 KD cells gained cobblestone morphology in monolayer culture and a complete loss of ability to invade in M/Col-I matrix as compared to the VECT control cell line (Fig 6B and 6C). To rule out any possible off-target effects of the short hairpin RNA (shRNA), we rescued the *Amigo2* expression by transducing the human *Amigo2* cDNA (or a VECT control) into the 4T1 AM2 KD (*Amigo2* knock-down rescue [AM2 KD-RE] and *Amigo2* knock-down control vector [AM2 KD-VECT], respectively) (Fig 6D). Reexpression of *Amigo2* restored both loss of cobblestone cell morphology and a restoration of 3D invasive properties (Fig 6E and 6F), demonstrating that *Amigo2* is required for the invasive behavior of K14+ cells. Interestingly, overexpression of *Amigo2* in the K14− cells (K14-AM2) did not affect 2D or 3D phenotype. Thus, while *Amigo2* is necessary for the maintenance of invasive behavior of 4T1 K14+ cells, it is not sufficient to promote invasive behavior in K14− mammary epithelial cell populations.

## Discussion

In this study, we generated and characterized novel reporter systems that combine the ability to track cells using a fluorescence protein expression with the more unique ability to selectively eliminate them using a toxin treatment. This strategy allowed us to select for K14+ mammary tumor cells and to demonstrate that they are indeed more invasive in culture and more tumorigenic and metastatic *in vivo*. By depleting K14+ cells *in vivo*, we demonstrated that K14+ cells are required for metastasis. Consistently, K14− and K8+ showed reduced invasion ability and a more organized epithelial-like monolayer than the K14+ population. Our studies also demonstrate the limits of differentiation plasticity, as K14− cells were unable to generate K14+ cells, whereas K14+ cells were capable of generating K14− or K14+ cells. To broaden the utility of the reporter system, we generated transgenic models and demonstrated that our promoters are active in the appropriate cellular compartment of the mammary gland and that K8.tGFP expression also matches endogenous K8 protein–expressing cells in lungs. Although the percentage of reporter-positive cells in our transgenic model is lower than the CRE-based systems previously reported, we would like to highlight that our platform is a “real-time” readout of the promoter activity, which creates opportunity to study plasticity of differentiation states. In the CRE-based lineage-tracing models, CRE is induced during the prolonged labeling periods (several days to a week), and the cells and their progenies are permanently marked irrespective of changes in its differentiation states. Furthermore, the combination of reporter and toxin gene expression *in vivo* makes these models valuable for all fields of biology involving K14 and K8 lineages. Since differentiation state plasticity plays a critical role in development and cancer, having the ability to eliminate cells that are present in a given differentiation state at the given time window is of significant importance for almost all types of carcinoma in multiple organs.

Orthotopic transplantation of K14− 4T1 cells resulted in formation of tumors that were smaller in size compared to K14+ or WT cells. Surprisingly, we did not observe any difference in the cell proliferation rates between K14− and K14+ cells in culture or *in vivo* (see S5A and S5B Fig). Although it is not clear why K14− cells form smaller tumors *in vivo*, there may be two possible explanations. First, albeit being transplanted into syngeneic background mice, K14+ cells may be better at evading immune systems compared to K14− cells and hence grow better *in vivo*. Second, a more provocative possibility relates to an interesting hypothesis proposed by a recent study that suggests that tumor growth rate can be substantially altered by changes in dispersal rate of cancer cells even in the absence of any change in doubling time [44]. Given that K14+ cells express mesenchymal state markers and demonstrate increased ability to migrate, it is possible that K14+ cells are more efficient in forming tumors *in vivo*. Additional experiments are needed to understand why K14− cells form smaller tumors compared to K14+ cells.

Previous studies demonstrate that K14− can become K14+ to lead the invasive front during metastasis of mouse mammary tumor virus polyomavirus middle T-antigen (MMTV-PyMT) luminal metastatic mouse model of breast cancer [12]. In our studies, we did not observe any significant impact on either primary tumor mass or metastatic lesions upon orthotopic injection of K14− treated with DT to deplete K14+ cells that could be generated from K14− cells. This observation suggests that plasticity of differentiation state may be a context-dependent phenomenon or that the K14− cells transition to a K14+ state without involving activation of the K14 reporter.

We demonstrate the utility of the platform to gain new molecular insights by comparing K14+ cells to K14− by RNA-seq and secretome analysis. We identified a number of genes differentially expressed or secreted. Many of these genes were previously reported to be associated with either bad prognosis or with invasion and/or metastatic phenotypes in human breast cancer, validating the power of the platform. In addition, we report the identification of *Amigo2* as a new mediator of invasion in breast cancer. Interestingly, *Amigo2* knockdown in 4T1 suppresses invasive behavior, but its overexpression in K14− cells was not sufficient to induce invasion, demonstrating that *Amigo2* is necessary but not sufficient for the invasiveness. *Amigo2* is an adhesion molecule that was first identified in 2003 and has been found to play a role in axon development [40]. It has been shown to be differentially expressed in gastric adenocarcinoma [45] and has been putatively identified as a possible metastasis-associated gene [46]. More recently, *Amigo2* has been found to play a role in endothelial and melanoma cells, as *Amigo2* regulates apoptosis [47–49]. *Amigo2* mediates adhesion of fibrosarcoma cells to the liver endothelium, resulting in colonization in the liver, which indicates another possible role for *Amigo2* in extravagation and colonization [50]. Our findings, together with the above observations, identify *Amigo2* as a potential therapeutic target for controlling metastasis.

## Materials and methods

### Ethics statement

All animal experiments were performed according to the protocol (AUP3218), which has been approved by the University Health Network Institutional Animal Care and Use Committee (IACUC).

### Antibodies and reagents

Primary antibodies to E-cadherin (clone 36) and β-catenin (clone 14) secondary antibody PE-Cy7 streptavidin and conjugated antibody CD24-APC (Clone M1/69) were purchased from BD Transduction Laboratories. Primary antibodies to fibronectin, β-actin (clone AC-15), Vimentin (for western blot, Clone VIM13.2), and α-tubulin (clone B-5-1-2) were purchased from Sigma-Aldrich. Primary antibody to GFP (NB100-1678) was from Novus Biologics, whereas to α-SMA (ab5694), CD31 (ab28364), GAPDH (ab8245), vimentin (immunofluorescence, ab92547), and Histone 3 Acetyl K27 (ab4729) were purchased from Abcam. Primary antibody to cleaved caspase-3 (CST 9661s) was purchased from Cell Signaling Technology. Primary antibody to Ki67 (RM-9106-S) was purchased from Thermo Scientific. Primary antibody to *Col6a1* (H-200), *Amigo2* (C-15), and β-1 integrin (M-106) were purchased from Santa Cruz Biotechnology. Primary antibodies to turboRFP (AB234) and turboGFP (AB513) were purchased from evrogen. K14 (AF 64) was purchased from Covance (PRB-155P). K8 was purchased from developmental studies hybridoma bank (Troma-1). Conjugated antibody CD49f-PerCP-Cy5.5 (Clone GoH3) and CD-44-Brilliant Violet 421 (Clone IM7) were purchased from Biolegend. Biotin-CD45 (Clone 30-F11), biotin-CD31 (Clone390), and biotin-Ter119 (Clone Ter119) (Lin- antibodies) were purchased from ebioscience.

Hoechst 33342 and 4’,6-diamidino-2-phenylindole (DAPI) were purchased from life technologies.

Secondary antibodies conjugated to Alexa Fluor 488 or Alexa Fluor 568 and Alexa Fluor 647 were purchased from Life Technologies. Secondary antibodies conjugated to peroxidase were purchased from Jackson ImmunoResearch Laboratories. DT purified from *Corynebacterium diphtheria* was purchased from Enzo Life Sciences. GCV was purchased from Sigma. Growth factor-reduced Matrigel and bovine Collagen I (BD Biosciences) were used for 3D culture, and Matrigel was used for orthotopic injection experiments.

### Construction of the reporters

The K14-Cre plasmid containing the human *KRT14* (*K14*) gene promoter followed by the rabbit β-globin intron [18] was kindly provided by Jos Jonkers (Netherlands Cancer Institute, Netherlands). The pEGFP-N1 vector was obtained from Clontech. The *K14* promoter–driven EGFP-P2A-DTR (K14-EPD) reporter construct was generated by multisite Gateway cloning (Life Technologies) according to the manufacturer’s instructions. The K14-β-globin entry clone was made by PCR amplification using sequence from K14-Cre as a template. The EPD entry clone was made by overlap extension PCR using EGFP and DTR cDNA templates and inserting P2A peptide coding sequence in between. The SV40pA entry clone was made by overlap extension PCR using oligonucleotides matching the late polyadenylation signal sequence (pA) of Simian virus 40 as a template. The three entry clones—K14-β-globin, EPD, and SV40pA—were recombined into a Gateway-compatible pBluescript KS+ destination vector to produce the final construct, pBS-K14β-EPD-SV40pA. The *K14* promoter–driven TurboRFP (K14-tRFP) reporter construct was generated in a similar manner, except using cDNA template for fast-maturating destabilized red (orange) fluorescent protein TurboRFP (tRFP, pTurboRFP-dest1; Evrogen) instead of EGFP, and DTR was replaced by herpes simplex virus TK. The K8 construct was also generated in a similar manner. The 3.5-kb sequence upstream the ATG codon of the murine K8 gene was obtained from the BAC clone RP23-254K21 (BACPAC Resources Center, Children’s Hospital Oakland Research Institute) using the forward primer 5′-GGTGGATCACTTGCCCCCTCCGTTTG-3′ and the reverse primer 5′-GGGACAGCGCCCAGCGAAGGCCC-3′ as previously done [13].

K8 promoter was followed by the rabbit β-globin intron [18], a fast-maturating and short-half-life turbo green fluorescent protein (tGFP, pTurboGFP-dest1 vector; Evrogen), a P2A followed by a DTR, and sv40polyA signal as described above. The two turbo constructs were also modified by adding a neomycin (for K14) and a hygromycin (for K8) resistance to allow selection.

SIN constructs were generated by PCR amplification from promoter to the end of the suicide gene. Primers were flanked with attB1 and attB2 sequence to allow gateway cloning into the destination SIN lentiviral vector pLBC2-B-RFCA.

### Cell culture, treatments, and generation of stable cell lines

4T1 mouse mammary tumor cells were maintained in DMEM (4.5 g/L glucose) supplemented with 10% fetal bovine serum (FBS; Life Technologies), 1X MEM nonessential amino acids (NEAA; Life Technologies), 100 U/ml penicillin, and 100 μg/ml streptomycin. If not specified, 3D cultures of 4T1 cells were grown embedded in a matrix of Matrigel or M/Col-I. Briefly, 96-well plates (BD Falcon) were coated with 30 μL of Matrigel or M/Col-I. The plate was put for 30 minutes at 37°C. Cells were added on top of the matrix in 20 ul of Matrigel or Matrigel:Collagen. After 30 minutes at 37°C, growing media supplemented with 5% Matrigel were added to the well. From day 3 to day 5, 3D structures were monitored for invasive protrusions. All cells were grown in a humidified atmosphere with 5% CO_2_ at 37°C. For DTR-mediated cell ablation in culture, cells were treated with 2.5 or 5 ng/ml DT (in 10 mM Tris-HCl pH 7.5, 1 mM Na_2_EDTA) for 2 to 7 days. For TK-mediated ablation, cells were treated with 1 or 5 ug/ml GCV for 5 to 7 days.

To generate stable cell lines, cells were cotransfected with the pBluescript-K14-EPD reporter construct and the pMSCV-zeo vector containing the *Sh ble* gene, using Lipofectamine 2000 (Life Technologies) according to the manufacturer’s instructions. After 48 hours, cells were replated in fresh growth medium containing 400 μg/ml Zeocin (Life Technologies), which was replenished every 4 days for a total of 16 days. Zeocin-resistant clones were pooled, replated, and expanded in the presence of Zeocin for an additional 7 days (pre-sort), and then EGFP-expressing cells were isolated by FACS (sort-1) using a MoFlo XDP High-Speed Cell Sorter (Beckman Coulter) equipped with a 488-nm laser. After 7 passages, these cells were subjected to a second round of FACS (sort-2) from which both the EGFP+ and EGFP− cells were collected. Cells were allowed to recover from sorting stress and then frozen down in batches. Except where indicated, all experiments using sorted cells were performed within 2 weeks after thawing. All cell lines were tested and found to be negative for mycoplasma and viral contamination by PCR. Turbo cell lines were generated in a similar manner, except selections were carried out with G418 (life technology) or Hygromicin (Roche) for K14 or K8, respectively. Cells were sorted with MoFlo Astrios EQ High Speed Cell Sorter (Beckman Coulter).

AM2 KD (mouse specific) and overexpression were achieved by lentiviral transduction of pLKO.1 shB7 (GTGTTCTCAGACACACCCTTT; TRCN0000182478) and pLX304 expressing full-length human *Amigo2* cDNA (Gene ID 347902, both kind gifts of Jason Moffat).

### Transgenic mice

Generation of the transgenic line was performed at the Centre for Phenogenomics. The K14.tRPT and K8.tGPD reporters were purified, and pronuclear microinjections into FVB/N zygotes produced from mating of superovulated females and stud males. Viable microinjected zygotes were transferred the same day into pseudopregnant CD-1 females for gestation and birth. The resultant pups were genotyped to identify founders. Every founder was mated with FVB/N mice and constituted 1 line. Every line has been screened for percentage of positive cells in the right compartment in regards to the mammary gland (see below Mammary gland isolation and flow cytometry analysis) and for the responsiveness to the depletion agents (GCV and DT). Based on the outcome, the best-performing lines (1 per K8.tGFP and 1 per K14.tRFP) were kept and propagated. DNA isolation and PCR genotyping were carried out with AccuStart II Mouse Genotyping Kit (Quanta Biosciences). Primer sequence for K14 mice were Fw AGCTTCATGTACGGCAGC, Rv GTACTTGGCCACAGCCATC; for K8 mice Fw CACGTGATGGGCTACGGC, Rv GTACTCCACGATGCCCAG.

### Mammary gland isolation and flow cytometry analysis

Single-cell mammary gland suspensions were generated from freshly isolated mammary glands of 11–21-week-old female mice by enzymatic digestion, adapting a previously described protocol [25]. Mammary glands were dissected using razor blades and digested in mouse Epicult-B media and 750 U/ml collagenase and 250 U/ml hyaluronidase for 2.5 hours at 37°C. Organoids obtained were quickly vortexed (3 pulses of 3 seconds each, medium speed) and resuspended in 0.8% ammonium chloride solution to lyse red blood cells. Organoids were further dissociated in 0.25% trypsin for 2 minutes, 5 mg/ml dispase 0.1mg/ml DNase I for 2 minutes and filtered through a 40-μm mesh to obtain single cells. All reagents were purchased from StemCell Technologies. Single cells were stained with Lin- antibody for exclusion and for CD24 and CD49f to identify luminal and basal population. DAPI was used for live/dead exclusion. Flow cytometry analyses were carried out with BD Biosciences Fortessa.

### RT-PCR

All PCR assays with reverse transcription were performed by using qScript cDNA SuperMix, (Quanta Biosciences). Real-time PCR was performed on a 7900HT system with TaqMan Universal Master Mix II (Life Technology). Taqman assays: *Amigo2* was purchased from IDT (Mm.PT.58.42252903); GAPDH was purchased from Applied Biosystems (#4352932).

### PCR

PCR was performed using genomic DNA isolated from cells lysed in ice-cold cell lysis buffer (10 mM Tris-HCl pH 8.0, 1 mM EDTA, 0.1% SDS). PCR reactions were carried out using 100 ng of DNA in a final volume of 25 μl. The primer sequences used are as follows: *monkey DTR* forward: 5′-AGCTCCTTCTGGCTGCAGTTCTTT-3′; *monkey DTR* reverse: 5′-TTTCCGAAGACATGGGTCCCTCTT-3′; *mouse GAPDH* forward: 5′-AACTTTGGCATTGTGGAAGGGCTC-3′; *mouse GAPDH* reverse: 5′-TGGAAGAGTGGGAGTTGCTGTTGA-3′.

### *In vitro* cell growth assays

Growth curves were generated from cells seeded at 3.5 × 10^5^ cells/dish in 60-mm dishes. At each time point, cells were trypsinized and counted using an automatic cell counter (TC10 Automated Cell Counter, Biorad).

MTT assays were performed on cells seeded in 96-well plates at 2,500 cells/well, in triplicate in 100 ul growing media. At each time point, cells were incubated with 0.5 mg/ml MTT (Thiazolyl Blue Tetrazolium Bromide; Sigma) for 4 hours at 37°C. Solubilization solution (10% SDS in 0.01 N HCL) was added (100 μl/well), and absorbance was measured at 560 nm using a spectrophotometer (BMG Labtech, FLUOstar Omega). Results were normalized at day 1.

Click-iT Plus EdU Alexa Fluor 647 Flow Cytometry Assay Kit was purchased from Thermo Scientific and used according to the standard protocol. The 4T1-generated cell lines were incubated with 10 μM EdU for 1 hour. DNA was stained with FxCycle Violet (Thermo Scientific). Results were acquired with 5 Laser LSR II (BD Biosciences) and analyzed by flowjo.

### Migration assay

Confluent monolayer was scratched with a p10 pipet tip, media were changed, and pictures were taken for time 0. After testing a series of time windows, we determined 6 hours to be the best time to assess differences. Therefore, 6 hours postscratch, pictures were taken. Pictures at time 0 and 6 hours were analyzed with ImageJ to calculate the area cells migrated (in pixels).

### Immunoblot analysis

Immunoblotting was performed using lysates from cells lysed in ice-cold RIPA buffer (50 mM Tris-HCl pH 7.5, 150 mM NaCl, 5 mM EDTA, 1% Triton X-100, 0.5% NaDOC, 0.1% SDS, and protease inhibitors). Protein concentrations of clarified cell lysates were determined using a bicinchoninic acid protein assay kit (Pierce, Thermo Scientific). Proteins were separated by SDS-PAGE and transferred to polyvinylidene fluoride membranes. Membranes were blocked in 5% milk, incubated with primary antibodies for 1 hour or overnight, and incubated with peroxidase-conjugated secondary antibodies for 1 hour minimum. Bound antibodies were detected using enhanced chemiluminescence (Pierce, Thermo Scientific). The protocol was adapted from [51].

### Immunostaining and image acquisition

Cells grown on glass coverslips were washed twice with PBS at room temperature, fixed with 4% formaldehyde in PBS for 12 minutes, permeabilized with 0.5% Triton X-100 in PBS for 10 minutes, and then blocked with 3% BSA in PBS for 30 minutes. Fixed cells were incubated with primary antibodies for 1 hour and incubated with fluorophore-conjugated secondary antibodies for 1 hour. Coverslips and slides were mounted with ProLong Gold antifade reagent (Life Technologies). Cells were imaged using a 60X Plan-Apochromat/1.40 oil or a 40X Apochromat LWD/1.15 water immersion objective on an inverted laser-scanning confocal microscope (C1si Confocal Microscope; Nikon), and images were captured using EZ-C1 Software (Nikon) or with Zeiss Axioimager M1 microscope with Plan-Apochromat 20×/0.8 air. The protocol was adapted from [51].

### Orthotopic mouse model

Female syngeneic BALB/c mice (Jackson Laboratory) were used for orthotopic injections at 8 weeks of age. The 4T1 cells (2.5 × 10^5^) in 20% Matrigel in a volume of 25 μl were injected into the abdominal mammary fat pad (#9) of anesthetized mice. Once primary tumors were palpable, they were measured 1–2 times per week using vernier calipers. For DTR-mediated cell ablation *in vivo*, mice were injected i.p. with 25 μg DT/kg body weight every other day for a total of 4 doses, beginning at day 7 after orthotopic cell injections. All mice were euthanized 4 weeks after cell injections. Tumors and lungs were collected and fixed in 10% neutral buffered formalin, dehydrated, and embedded in paraffin for tissue sectioning. Tumor measurements and analysis of lung metastasis were derived from multiple experiments using a total of 4–8 mice per group.

### Histology and quantification of lung metastases

H&E staining of lung sections was performed by the Campbell Family Institute of Breast Cancer Research Histology Core Unit. Metastases were identified by histopathological examination of images of H&E-stained sections acquired on a NanoZoomer 2.0-HT digital slide scanner (Hamamatsu). Lung metastasis was quantified by measuring the average percentage of area occupied by the tumor (% area = [area tumor / area total tissue] × 100%) in 5 sections (5-μm thickness) cut at 200-μm intervals using ImageJ software (National Institutes of Health).

### Immunohistochemistry and image acquisition

For immunohistochemistry, 5-μm paraffin sections were deparaffinized in xylene and rehydrated in a graded ethanol series. Sections were boiled in citrate buffer (10 mM sodium citrate pH 6.0) or Trilogy (Cell Marque) for 5 minutes in a microwave oven and allowed to cool at room temperature for 1 hour. Sections were blocked 5% BSA 0.5% Tween-20 in PBS for 1 hour, then incubated with primary antibodies overnight at 4°C. Sections were incubated with fluorophore-conjugated secondary antibodies for 1 hour and with Hoechst or DAPI to stain nuclei. Slides were mounted with Vectashield Mounting Medium (Vector Laboratories). Sections were imaged using a 40X Super Apochromat/0.95 air objective on an inverted laser-scanning confocal microscope (FluoView FV1000; Olympus), and images were captured using FV10 Software (Olympus) or with Zeiss Axioimager M1 microscope with Plan-Apochromat 20x/0.8 air and Plan-Apochromat 40x/1.3 Oil DIC objective lenses. Only for Fig 1I, the images were acquired with Zeiss LSM 880 Inverted Laser Scanning Microscope with Plan-Apochromat 10x/0.45 and Plan-Apochromat 20x/0.8 air objective lenses. Postprocessing was carried out with ImageJ and photoshop. Acquisition and postprocessing parameter were kept constant across the figures that are directly compared. Only for Fig 1I, the bright field contrast was modified for 1 of the pictures because of different shades in different fields due to the irregularity of the Mat/Col-I matrix.

The single immunohistochemistry antigen labeling of the paraffin-embedded murine samples was performed with Ki-67 and cleaved caspase 3 antibodies. The paraffin sections were deparaffinized and rehydrated, followed by antigen retrieval using sodium citrate buffer (pH 6). The sections were then incubated with 3% hydrogen peroxide to block endogenous peroxidase activity for 10 minutes at room temperature. After 3 washes with TBS, the sections were incubated with 2% bovine serum albumin (Jackson ImmunoResearch Lab Inc, West Grove, PA) for 1 hour at room temperature. Slides were then incubated separately with rabbit anti-Ki67 (RM-9106-S) and rabbit anti-cleaved caspase 3 (CST 9661s) overnight at 4°C. The slides were washed 3 times and incubated with goat anti-rabbit HRP polymer (1:1, Abcam ab214880 lot: GR3196509-1) for 2 hours at room temperature. Samples were washed thrice and then developed using the ImmPACT DAB Peroxidase (HRP) Substrate kit (Vector Laboratories, SK-4105). Samples were counterstained with hematoxylin and then dehydrated before mounting the slides with permount.

Ki-67 and CC3 immunohistochemistry expression were quantified using an automated imaging analysis software program, Definiens Tissue Studio version 4.4.2 (Munich, Germany), which produced a 0%–100% continuous estimate of expression. Nuclear reactivity was quantified for Ki-67, while cytoplasmic reactivity was quantified for CC3. The percentage of positive reactivity was calculated by dividing the number of positive nuclei/cells by the total number of nuclei/cells detected on each whole slide image.

### Secretome analysis

CM from cells cultured for at least 24 hours in phenol red- and FBS-free media were collected, cell debris was removed by centrifugation, and the clarified CM were concentrated using centrifugal filter units (Amicon Ultracel MWCO 3 kDa). Secreted proteins were acetone-precipitated and resuspended in 8 M urea and 50 mM ammonium bicarbonate and reduced and alkylated with 10 mM dithiotreitol and 50 mM iodoacetamide, respectively. Samples were diluted with ammonium bicarbonate pH 8.5 to 1.5 M urea and digested with proteomics-grade trypsin (Promega). Multidimensional protein identification technology (MudPIT) analysis was performed as previously described [52]. Briefly, a 4-cycle 2D chromatography sequence was set up, and peptides were separated based on charge by strong cation exchange resin and on hydrophobicity by C18 reverse-phased resin. Samples were run in triplicate on a hybrid ion trap-Orbitrap mass spectrometer (LTQ Orbitrap XL; Thermo Scientific). Three biological replicates per condition were analyzed. Spectral counting (SpC) was used as a measure of protein abundance. The SpCs for peptides corresponding to a protein were normalized against the total number of spectra for a given MudPIT sequence, averaged over the triplicates. An arbitrary value of 0.1 was added to every SpC to avoid division by 0. The relative abundance of each protein was calculated as the ratio of averaged normalized SpCs in the CM from K14+ versus K14− cells.

### RNA-Seq analysis

Total RNA was purified and DNAse-treated using the RNeasy Mini Kit (Qiagen). RNA quality (RNA integrity number > 9) and quantity were measured on a Bioanalyzer (RNA Nano kit; Agilent). The NuGEN Ovation RNA-Seq V2 protocol was carried out on 100 ng of total RNA. In brief, RNA was reverse transcribed using oligo-d(T) primers and random hexamers to generate cDNA, which was followed by SPIA (NuGEN) linear amplification. The cDNA was fragmented by sonication (Covaris E-series ultrasonicator) according to the manufacturer’s instructions to yield a target fragment size of 200 bp. The fragmented cDNA was subsequently processed according to the Illumina mRNA sequencing sample preparation guide (end repair, A-tailing, and ligation of sequencing adapters). The ligation products were run on a precast 4% NuSieve Agarose gel (Lonza), stained with SYBR Gold (Life Technologies), and selected for the 200–300-bp range. The size-selected cDNA was gel purified, PCR-amplified, and quantified using a Bioanalyzer (DNA 1000 kit; Agilent). Each sample was sequenced using an Illumina GAII sequencer on a single lane of a flow cell generating 50 nt single-end (SE) reads. The sequencing reads were aligned to the mouse genome (mm9) using TopHat software (v2.0.4), restricting only uniquely mapped reads to the genome. Cuffdiff software (v2.0.2) was employed to find significant changes in transcript expression between two conditions. Gene-level expression measurements are reported in fragments per kilobase per million reads (FPKM). Genes were designated as differentially expressed between two conditions if the false discovery rate (FDR)-corrected *p* < 0.05 for differential expression, fold change > log2(2), and FPKM > 5 in at least one condition.

### H3K27ac chromatin immunoprecipitation

One million sorted cells per condition (2 biological replicates) were fixed by immersion in 1 ml of 1% formaldehyde/PBS for 10 minutes at room temperature with rotation. Following fixation, cells were spun down at 3,000 rpm for 3 minutes, resuspended in 500 μL ice-cold PBS/BSA, spun down again, and resuspended in 500 μL ice-cold PBS. Cells were then spun a final time and resuspended in 350 μL of cell lysis buffer (1% SDS, 10 mM EDTA, 50 mM Tris-HCl pH 8.1). Cells were then sonicated on high setting for 30 cycles of 30 seconds on/30 seconds off using a Diagenode Bioruptor 300. Insoluble cell debris was removed by centrifugation at 4°C for 15 minutes at 15,000 rpm. Then, 20 uL of each sample was removed and set aside as an input sample, while 320 uL was added to 1.6 mL of cold dilution buffer (1% Triton X-100, 2 mM EDTA, 150 mM NaCl, 20 mM Tris-HCl pH8.1).

Per ChIP, 10 uL each of protein A and protein G Dynabeads (Thermo Fisher Scientific, cat#10002D and 10004D, respectively) were washed 3 times in cold PBS/BSA (5 mg/mL) and resuspended in 300 uL cold PBS/BSA, and 3 ug of H3K27ac antibody was added. The antibody/bead mixture was then incubated at 4°C for 6 hours with rotation, washed twice with cold PBS/BSA, and resuspended in 100 uL cold dilution buffer. The antibody/bead solution was then added to the processed chromatin sample and incubated overnight at 4°C with rotation. The following day, immunoprecipitated chromatin was washed 3 times with cold washing RIPA buffer (1% NP-40, 0.7% sodium deoxycholate, 50 mM HEPES, 1 mM EDTA), twice with TE buffer, and resuspended in 100 uL decrosslinking buffer (1% SDS, 0.1 M NaHCO_3_). Samples were then incubated for ≥6 hours at 65°C, and DNA was purified using a Qiagen MinElute kit. The protocol was adapted from [53].

### Library preparation, sequencing, alignment of ChIPseq reads, and peak calling

Sequencing libraries were prepared using 0.5–10 ng of ChIP or input DNA with the Rubicon Thruplex FD kit, using the manufacturer’s recommended protocol, and were size selected in the range of 240–360 bp using a Caliper LabChIP XT DNA 750 kit (Perkin-Elmer). Size-selected libraries were then sequenced on an Illumina HiSeq 2000 with SE 50-bp reads. Casava-processed reads were aligned to the mouse genome (mm9) using Bowtie 2.0 software (v2.0.5) with default parameters. Duplicate reads were removed using Samtools software (v0.1.18). Peaks were called using MACS1.4 using default settings. The protocol was adapted from [52].

### Statistical analysis

The specific statistical tests used are indicated in the figure legends.

Briefly, statistical significance for tumor diameter was assessed by one-way ANOVA followed by Newman-Keuls multiple comparisons posttest. For final tumor masses (no DT experiment) and lung metastatic area, one-way ANOVA was followed by Tukey’s multiple comparisons posttest to assess differences between the means.

Linear regression analysis was used to assess no significant correlation between tumor mass and lung metastatic load. Kaplan-Meier plot significance was calculated by log-rank test. All the other *p*-values in this study were calculated by *t* test.

## Supporting information

S1 Fig (A) IF shows detection of endogenous keratin-14 and reporter-generated fluorescence protein (GFP) in K14.GFP− monolayer (scale bar 20 μm). (B) PCR analysis of genomic DNA from control and K14.GFP 4T1 cell lines confirming the presence of the transgene. (C) shows the dot plot for EdU incorporation on DNA staining analysis for K14.GFP+ and K14.GFP− cells. (D) shows the MTT assay of K14.GFP+; K14.GFP− and WT. Graphs show the mean ± SEM of 4 independent experiments. (E) Late passage K14.GFP+ cells monolayers were treated with DT (2.5 ng/ml) for 48 hours and then monitored for GFP expression by flow cytometry. The data shown are means ± SEM from 4 independent experiments. DT, diphtheria toxin; EdU, 5-Ethynyl-2´-deoxyuridine; GFP, green fluorescent protein; IF, immunofluorescence; MTT, 3-(4,5-dimethylthiazol-2-yl)-2,5-diphenyltetrazolium bromide.
(TIF)

S2 Fig (A) Representative images of H&E-stained tumors from mice injected with 4T1 K14.GFP reporter cell lines; scale bar 100 μm. (B) Representative images IHC for Ki67 (upper panel) and CC3 (lower panel); scale bar 50 μm. (C) Representative images of fluorescent IHC staining for endothelial marker CD31 with quantifications, shown are means of number of vessel/field of view (40×) ± STD; scale bar 20 μm. H&E, hematoxylin and eosin; IHC, immunohistochemistry.
(TIF)

S3 Fig (A) Fluorescent IHC detecting K14 and GFP on primary tumors generated from K14.GFP− cell lines either DT− or DT treated (DT+); scale bar 40 μm. (B) Same staining as described in (A) was carried out on metastatic lungs of mice injected with the indicated cell line; scale bar 20 μm. (A) and (B) DT+, the mice were injected i.p. with DT (25 mg/kg) on days 7, 9, 11, and 13. DT, diphtheria toxin; GFP, green fluorescent protein; IHC, immunohistochemistry; i.p., intraperitoneally; K, cytokeratin.
(TIF)

S4 Fig (A) Stably transfected K14.tRPT and K8.tGPD reporter cells were sorted (*t* = 0) by FACS and monitored for percentage of tRFP- and tGFP-expressing cells by flow cytometry for 30 days. (B) shows K8+ cell line stained for tGFP and K8. (C) shows K14+ (upper panels) and K14− (lower panels) stained for K14 or detection of endogenous tRFP signal. All IFs were counterstained with DAPI and have a merge of all channels. Scale bars 20 μm. (D) Quantification of migration assay for K14+ or K14− cell lines. Graph shows the mean ± SEM of 4 independent experiments, *p* < 0.0001 by unpaired *t* test. DAPI, 4’,6-diamidino-2-phenylindole; FACS, fluorescence-activated cell sorting; IF, immunofluorescence; K, cytokeratin; K8.tGPD, keratin-8 promoter followed by turbo green fluorescent protein and diphtheria toxin receptor; K14.tRPT, keratin-14 promoter followed by a turbo red fluorescent protein and herpes simplex virus thymidine kinase; tGFP, turbo green fluorescent protein; tRFP, turbo red fluorescent protein.
(TIF)

S5 Fig (A) shows the dot plot for EdU incorporation on DNA staining analysis for K14+ and K14−. Quantification of the cell cycle phases is given in the column bar as percentage of cells. Shown is the mean ± SD of triplicates of 1 representative experiment. (B) shows the MTT assay of K14+ and K14−. Graphs show the mean ± SEM of 4 independent experiments. (C) K14+ and K8+ cells were treated with either DT (2.5 ng/ml), GCV (1 μg/ml), or media and then analyzed by flow cytometry. Dot plots show the percentage of reporter-positive cells after treatments. DT, diphtheria toxin; EdU, 5-Ethynyl-2´-deoxyuridine; GCV, ganciclovir; MTT, 3-(4,5-dimethylthiazol-2-yl)-2,5-diphenyltetrazolium bromide.
(TIF)

S6 Fig (A) Fluorescent IHC was performed for vimentin, β-catenin, and GFP counterstained with DAPI on primary tumors generated from the either K14.GFP+ or K14.GFP− cell lines. Squares indicate regions that have been magnified 3×. (B) K14.GFP+ (upper panel) and K14.GFP− (lower panel); scale bars 50 μm. DAPI, 4’,6-diamidino-2-phenylindole; GFP, green fluorescent protein; IHC, immunohistochemistry.
(TIF)

S7 Fig (A) IF shows detection of E-cadherin immunostaining (upper) and GFP expression (lower) of 4T1 K14.GFP+ and K14.GFP− cell lines; scale bar 20 μm. (B) Fluorescent IHC shows detection of E-cadherin in tumors derived from either K14.GFP+ or K14.GFP− cell lines; scale bar 20 μm. (C and D) Upper panels show the dot plots and percentage of reporter positive or negative for K14.tRFP (C) or K14.GFP (D) cell lines. The lower panel shows the percentage of CD24 and CD44 positive cells for either total population, reporter-positive or reporter-negative fraction. GFP, green fluorescent protein; K, cytokeratin; IF, immunofluorescence; IHC, immunohistochemistry; tRFP, turbo red fluorescent protein.
(TIF)

S8 Fig (A) Cells from mammary glands for either WT, K8.tGPD, or K14.tRPT mouse were analyzed by flow cytometry, and percentage of reporter-positive cells for stroma, basal, and luminal compartments are shown. The first dot plot shows the total population per compartment, whereas the second shows only the cells that are positive for the reporters. Gates were set based on the negative control, and percentages are given in a color-code manner for the reporter-positive cells. (B) Table summarizing results of A for multiple mice. Percentage of reporter-positive cells in the right compartment. (C) H&E images of liver from either control (WT) or K8.tGPD mouse after exposure to high-dose DT. Scale bar 50μm. (D) H&E images of MG of either control (WT) or K14.tRPT mouse after exposure to low-dose GCV. Scale bar 100 μm. GCV, ganciclovir; H&E, hematoxylin and eosin; K8.tGPD, keratin-8 promoter followed by turbo green fluorescent protein and diphtheria toxin receptor; K14.tRPT, keratin-14 promoter followed by a turbo red fluorescent protein and herpes simplex virus thymidine kinase; MG, mammary gland; WT, wild-type.
(TIF)

S9 Fig (A) Shows the scatter plots in which each dot is a peak of H3K27ac plotted according to the ChIPseq signal in the specified replicate (“rep”) of the indicated cell line. The red dots correspond to the peak at the Amigo2 promoter. (B) HCC1143 human breast cancer cell line expressing the SIN lentiviral K14.tRPT or K8.tGPD reporter were sorted for the constitutive expressed tBFP and subsequently sorted for tRFP (upper panels) or tGFP (lower panel). Amigo2, amphoterin-induced protein 2; ChIPseq, chromatin immunoprecipitation sequencing; H3K27ac, histone 3 lysine 27; K8.tGPD, keratin-8 promoter followed by turbo green fluorescent protein and diphtheria toxin receptor; K14.tRPT, keratin-14 promoter followed by a turbo red fluorescent protein and herpes simplex virus thymidine kinase; SIN, self-inactivating; tBFP, blue fluorescent protein; tRFP, turbo red fluorescent protein.
(TIF)

S10 Fig Kaplan-Meier plot in all (A) breast cancer (“all BC”), (B) ER− and ER+, and (C) HER2 amplification positive (HER2+) and negative (HER2−) breast cancer shows relationship between Amigo2 expression and relapse-free survival. Amigo2, amphoterin-induced protein 2; ER, estrogen receptor; HER2 human epidermal growth factor receptor 2.
(TIF)

S1 Data The file includes the raw data for the secretome analysis. (XLSX)

S2 Data The file includes the raw data for RNA-seq analysis. RNA-seq, RNA sequencing.
(XLSX)

S3 Data The file contains the numerical values used to generate all graphs included in the manuscript. (XLSX)

S4 Data Representative dot plot showing the gating strategy for Fig 4A and S8A Fig. (TIF)

S5 Data Representative dot plot showing the gating strategy for (A) S1C and S5A Figs, (B) S5C Fig, (C) S7C Fig, (D) S7D Fig, and (E) S9B Fig.
(TIF)

S6 Data Raw data for H3K27Ac Chip for K14− first replicate. Chip, chromatin immunoprecipitation; H3K27Ac, histone 3 lysine 27; K, cytokeratin.
(GZ) Click here for additional data file.

S7 Data Raw data for H3K27Ac Chip for K14− second replicate. Chip, chromatin immunoprecipitation; H3K27Ac, histone 3 lysine 27; K, cytokeratin.
(GZ) Click here for additional data file.

S8 Data Raw data for H3K27Ac Chip for K14+ first replicate. Chip, chromatin immunoprecipitation; H3K27Ac, histone 3 lysine 27; K, cytokeratin.
(GZ) Click here for additional data file.

S9 Data Raw data for H3K27Ac Chip for K14+ second replicate. Chip, chromatin immunoprecipitation; H3K27Ac, histone 3 lysine 27; K, cytokeratin.
(GZ) Click here for additional data file.

## Abbreviations

3D: three-dimensional
AM2 KD: *Amigo2* knock-down
AM2 KD-RE: *Amigo2* knock-down rescue
AM2 KD-VECT: *Amigo2* knock-down control vector
Amigo2: amphoterin-induced protein 2
Areg: amphiregulin
BL: basal-like
Car9: carbonic anhydrase 9
CRE: causes recombination
Cav1: caveolin 1
Cd74: HLA-DR antigens-associated invariant chain
ChIPseq: chromatin immunoprecipitation sequencing
CM: conditioned media
Col6a1: Collagen VI subunit A
DAPI: 4’,6-diamidino-2-phenylindole
DT: diphtheria toxin
DTR: diphtheria toxin receptor
EdU: 5-Ethynyl-2´-deoxyuridine
EF-1α: *elongation* *factor 1α*
EGFP: enhanced green fluorescent protein
EMT: epithelial–mesenchymal transition
ER: estrogen receptor
ERK1/2: extracellular signal-regulated kinase ½
FACS: fluorescence-activated cell sorting
FDR: false discovery rate
FPKM: fragments per kilobase per million reads
FVB: Friend leukemia virus B
GCV: ganciclovir
GFP: green fluorescent protein
Gpnmb: glycoprotein nonmetastatic B
H3K27Ac: histone 3 lysine 27
H&E: hematoxylin and eosin
HER2: human epidermal growth factor receptor 2
IACUC: Institutional Animal Care and Use Committee
i.p.: intraperitoneally
K: cytokeratin
K8.tGPD: keratin-8 promoter followed by turbo green fluorescent protein and diphtheria toxin receptor
K14-AM2: cytokeratin 14 negative *Amigo2*-overexpressing cell
K14.tRPT: keratin-14 promoter followed by a turbo red fluorescent protein and herpes simplex virus thymidine kinase
LTR: long terminal repeat
M/Col-I: 1:1 mixture of Matrigel/Collagen-I
MMTV-PyMT: mouse mammary tumor virus polyomavirus middle T-antigen
Mt2: metallothionein-2
MTT: 3-(4,5-dimethylthiazol-2-yl)-2,5-diphenyltetrazolium bromide
MudPIT: multidimensional protein identification technology
NEAA: nonessential amino acids
pA: polyadenylation signal sequence
Pmp22: peripheral myelin protein 22
RNA-seq: RNA sequencing
SE: single-end
shRNA: short hairpin RNA
SLPI: secretory leukocyte peptidase inhibitor
SpC: spectral counting
tBFP: turbo blue fluorescent protein
tGFP: turbo green fluorescent protein
TK: thymidine kinase
tRFP: turbo red fluorescent protein
VECT: control vector
WT: wild-type
