# Supplementary figures and images for: Reporters to mark and eliminate basal or luminal epithelial cells in culture and in vivo
Source: PLoS Biol. 2018 Jun 20;16(6):e2004049. doi: 10.1371/journal.pbio.2004049 (PMC6042798; doi:10.1371/journal.pbio.2004049)

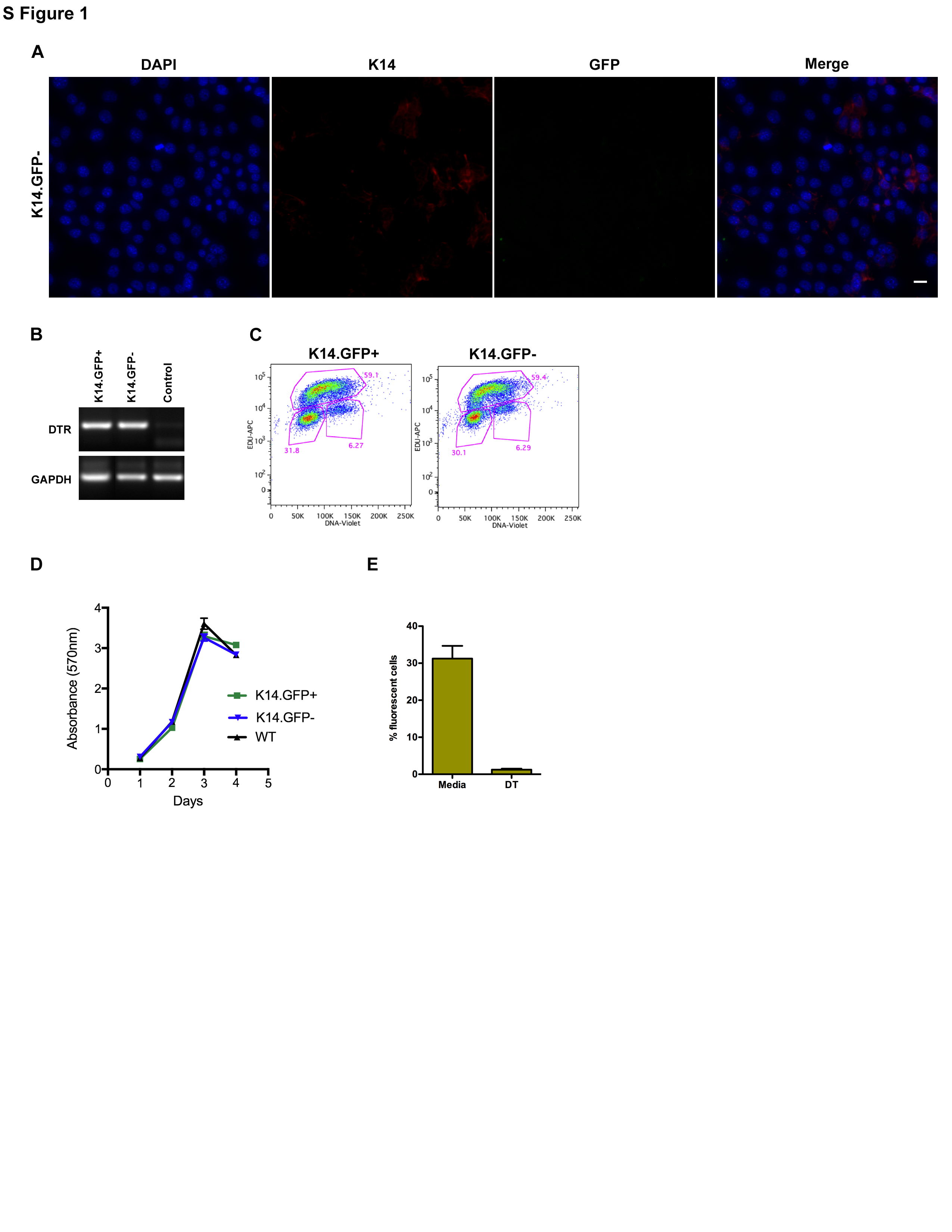

Supplement: S1 Fig — (A) IF shows detection of endogenous keratin-14 and reporter-generated fluorescence protein (GFP) in K14.GFP− monolayer (scale bar 20 μm). (B) PCR analysis of genomic DNA from control and K14.GFP 4T1 cell lines confirming the presence of the transgene. (C) shows the dot plot for EdU incorporation on DNA staining analysis for K14.GFP+ and K14.GFP− cells. (D) shows the MTT assay of K14.GFP+; K14.GFP− and WT. Graphs show the mean ± SEM of 4 independent experiments. (E) Late passage K14.GFP+ cells monolayers were treated with DT (2.5 ng/ml) for 48 hours and then monitored for GFP expression by flow cytometry. The data shown are means ± SEM from 4 independent experiments. DT, diphtheria toxin; EdU, 5-Ethynyl-2´-deoxyuridine; GFP, green fluorescent protein; IF, immunofluorescence; MTT, 3-(4,5-dimethylthiazol-2-yl)-2,5-diphenyltetrazolium bromide. (TIF) [file pbio.2004049.s001.tif]

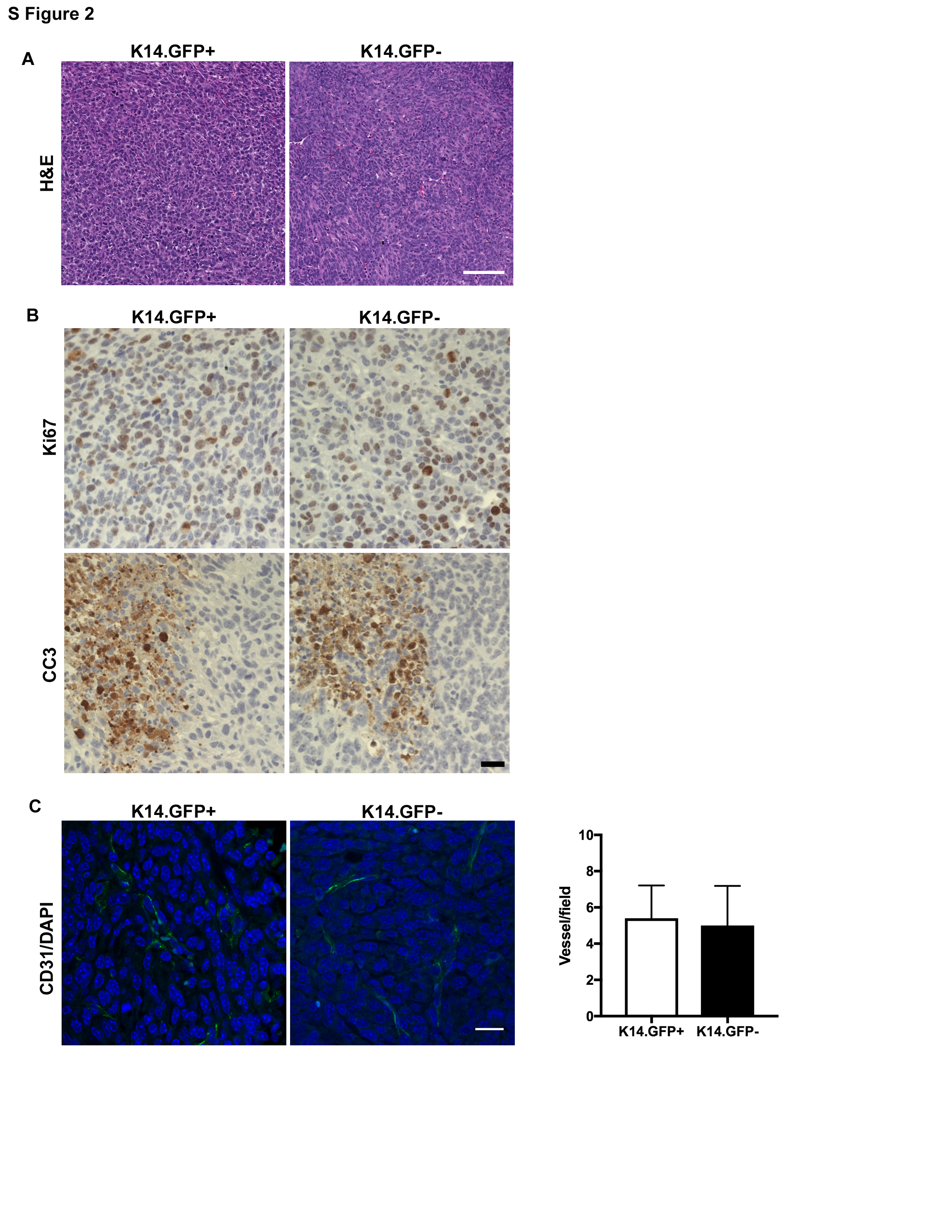

Supplement: S2 Fig — (A) Representative images of H&E-stained tumors from mice injected with 4T1 K14.GFP reporter cell lines; scale bar 100 μm. (B) Representative images IHC for Ki67 (upper panel) and CC3 (lower panel); scale bar 50 μm. (C) Representative images of fluorescent IHC staining for endothelial marker CD31 with quantifications, shown are means of number of vessel/field of view (40×) ± STD; scale bar 20 μm. H&E, hematoxylin and eosin; IHC, immunohistochemistry. (TIF) [file pbio.2004049.s002.tif]

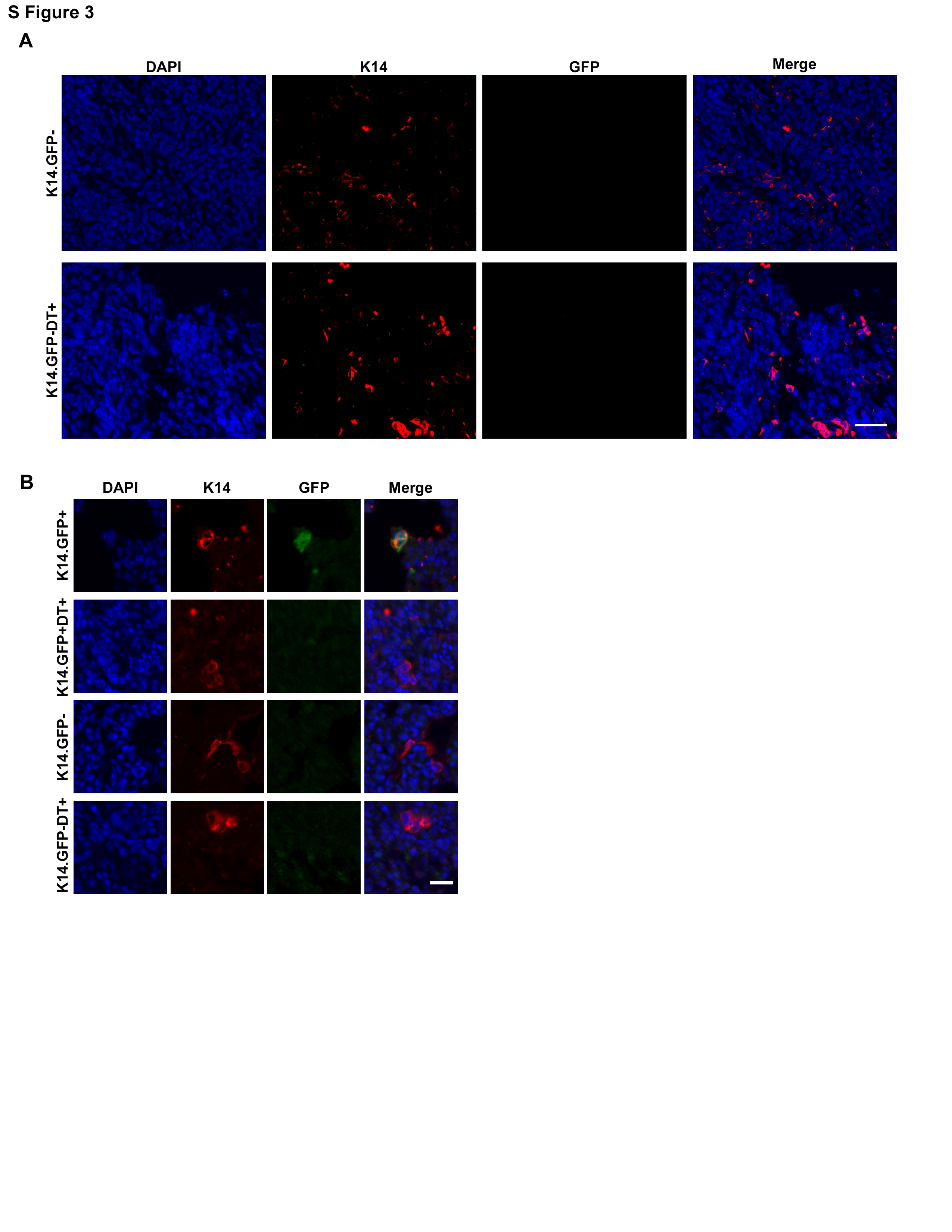

Supplement: S3 Fig — (A) Fluorescent IHC detecting K14 and GFP on primary tumors generated from K14.GFP− cell lines either DT− or DT treated (DT+); scale bar 40 μm. (B) Same staining as described in (A) was carried out on metastatic lungs of mice injected with the indicated cell line; scale bar 20 μm. (A) and (B) DT+, the mice were injected i.p. with DT (25 mg/kg) on days 7, 9, 11, and 13. DT, diphtheria toxin; GFP, green fluorescent protein; IHC, immunohistochemistry; i.p., intraperitoneally; K, cytokeratin. (TIF) [file pbio.2004049.s003.tif]

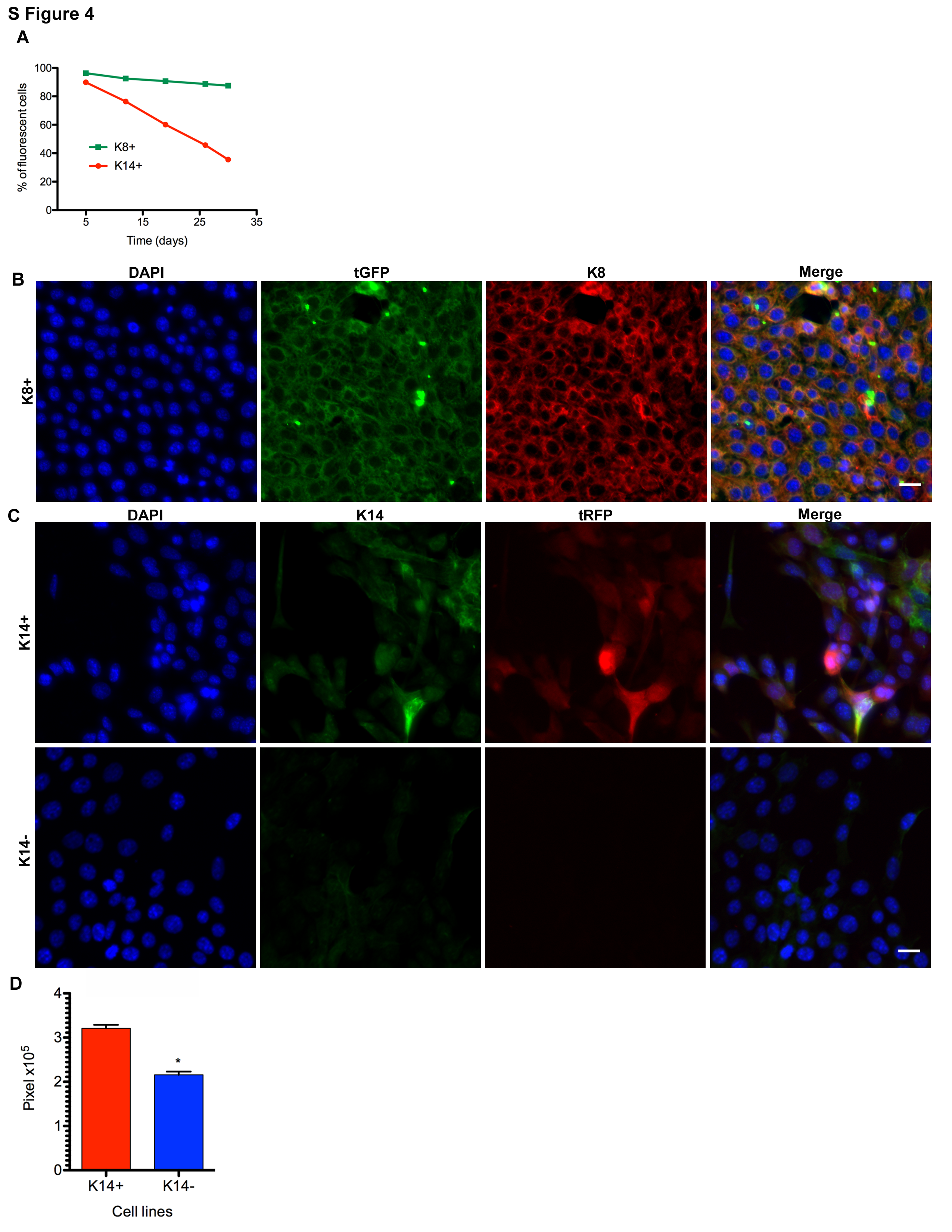

Supplement: S4 Fig — (A) Stably transfected K14.tRPT and K8.tGPD reporter cells were sorted (t = 0) by FACS and monitored for percentage of tRFP- and tGFP-expressing cells by flow cytometry for 30 days. (B) shows K8+ cell line stained for tGFP and K8. (C) shows K14+ (upper panels) and K14− (lower panels) stained for K14 or detection of endogenous tRFP signal. All IFs were counterstained with DAPI and have a merge of all channels. Scale bars 20 μm. (D) Quantification of migration assay for K14+ or K14− cell lines. Graph shows the mean ± SEM of 4 independent experiments, p < 0.0001 by unpaired t test. DAPI, 4’,6-diamidino-2-phenylindole; FACS, fluorescence-activated cell sorting; IF, immunofluorescence; K, cytokeratin; K8.tGPD, keratin-8 promoter followed by turbo green fluorescent protein and diphtheria toxin receptor; K14.tRPT, keratin-14 promoter followed by a turbo red fluorescent protein and herpes simplex virus thymidine kinase; tGFP, turbo green fluorescent protein; tRFP, turbo red fluorescent protein. (TIF) [file pbio.2004049.s004.tif]

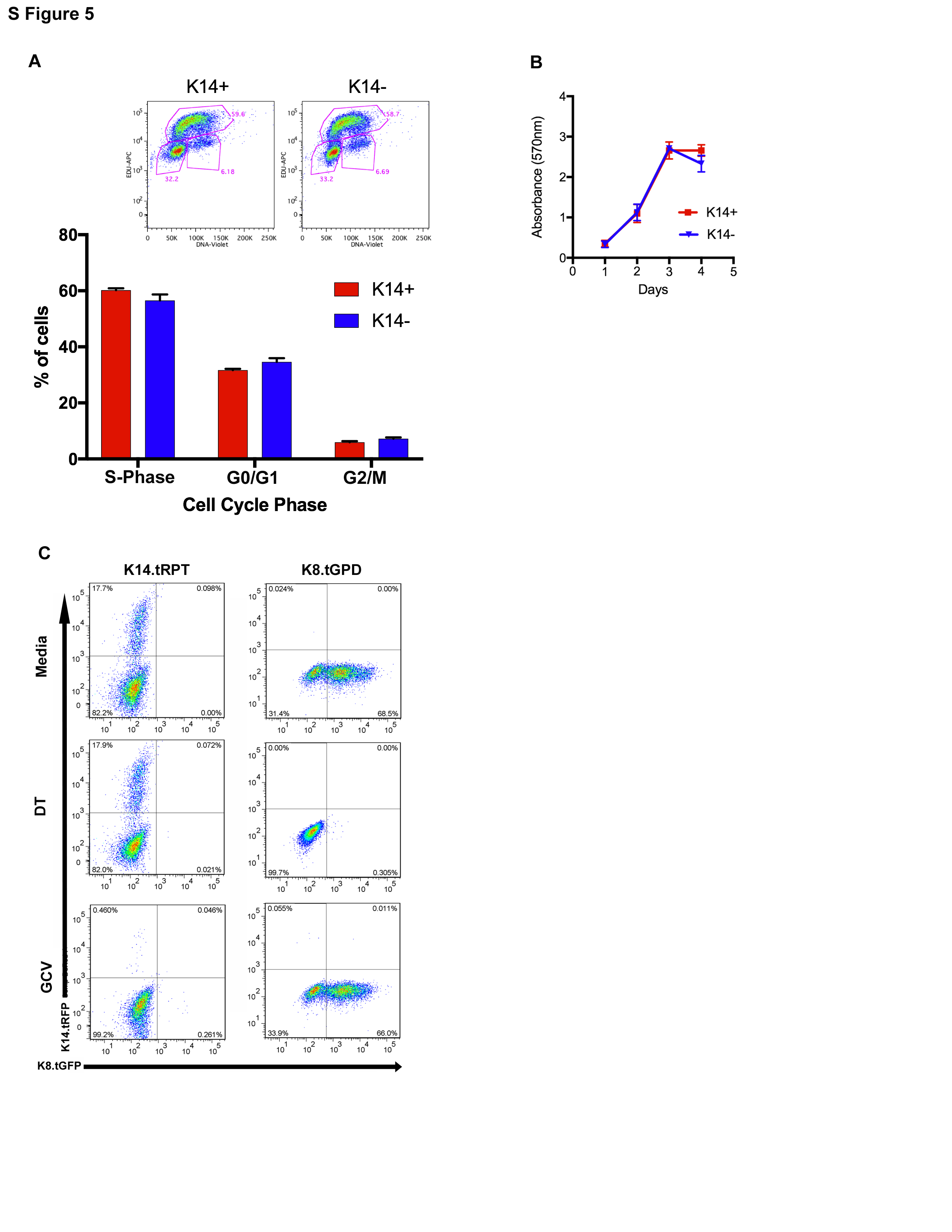

Supplement: S5 Fig — (A) shows the dot plot for EdU incorporation on DNA staining analysis for K14+ and K14−. Quantification of the cell cycle phases is given in the column bar as percentage of cells. Shown is the mean ± SD of triplicates of 1 representative experiment. (B) shows the MTT assay of K14+ and K14−. Graphs show the mean ± SEM of 4 independent experiments. (C) K14+ and K8+ cells were treated with either DT (2.5 ng/ml), GCV (1 μg/ml), or media and then analyzed by flow cytometry. Dot plots show the percentage of reporter-positive cells after treatments. DT, diphtheria toxin; EdU, 5-Ethynyl-2´-deoxyuridine; GCV, ganciclovir; MTT, 3-(4,5-dimethylthiazol-2-yl)-2,5-diphenyltetrazolium bromide. (TIF) [file pbio.2004049.s005.tif]

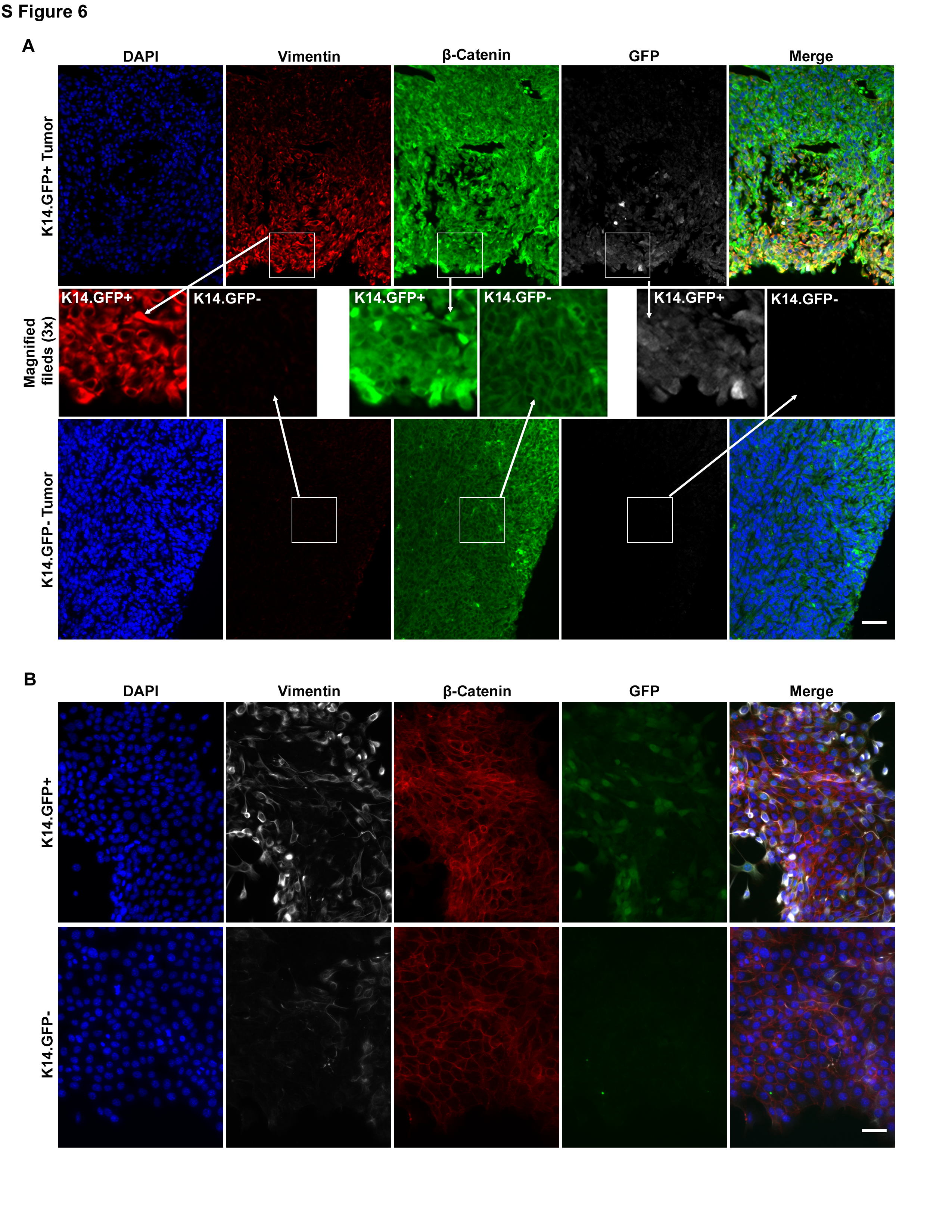

Supplement: S6 Fig — (A) Fluorescent IHC was performed for vimentin, β-catenin, and GFP counterstained with DAPI on primary tumors generated from the either K14.GFP+ or K14.GFP− cell lines. Squares indicate regions that have been magnified 3×. (B) K14.GFP+ (upper panel) and K14.GFP− (lower panel); scale bars 50 μm. DAPI, 4’,6-diamidino-2-phenylindole; GFP, green fluorescent protein; IHC, immunohistochemistry. (TIF) [file pbio.2004049.s006.tif]

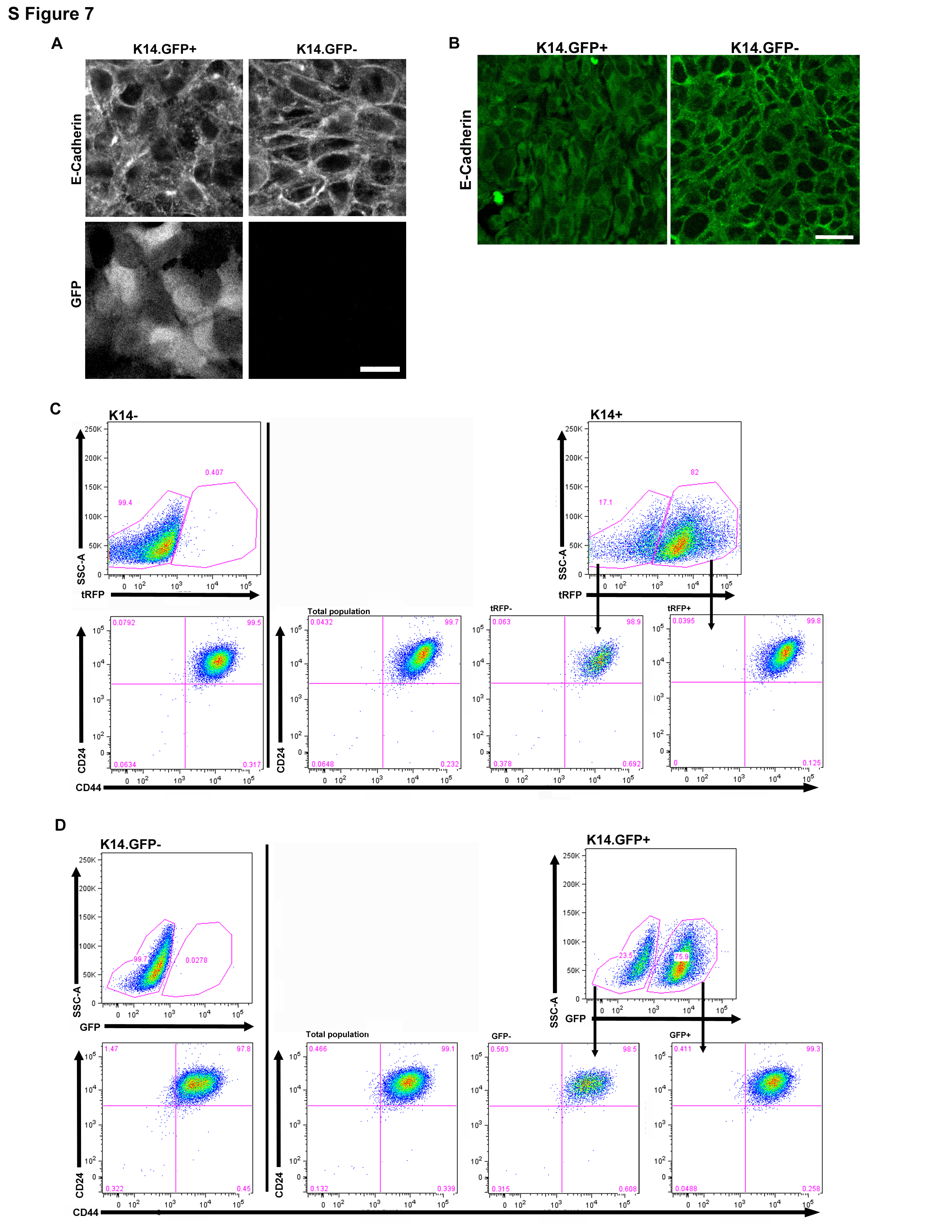

Supplement: S7 Fig — (A) IF shows detection of E-cadherin immunostaining (upper) and GFP expression (lower) of 4T1 K14.GFP+ and K14.GFP− cell lines; scale bar 20 μm. (B) Fluorescent IHC shows detection of E-cadherin in tumors derived from either K14.GFP+ or K14.GFP− cell lines; scale bar 20 μm. (C and D) Upper panels show the dot plots and percentage of reporter positive or negative for K14.tRFP (C) or K14.GFP (D) cell lines. The lower panel shows the percentage of CD24 and CD44 positive cells for either total population, reporter-positive or reporter-negative fraction. GFP, green fluorescent protein; K, cytokeratin; IF, immunofluorescence; IHC, immunohistochemistry; tRFP, turbo red fluorescent protein. (TIF) [file pbio.2004049.s007.tif]

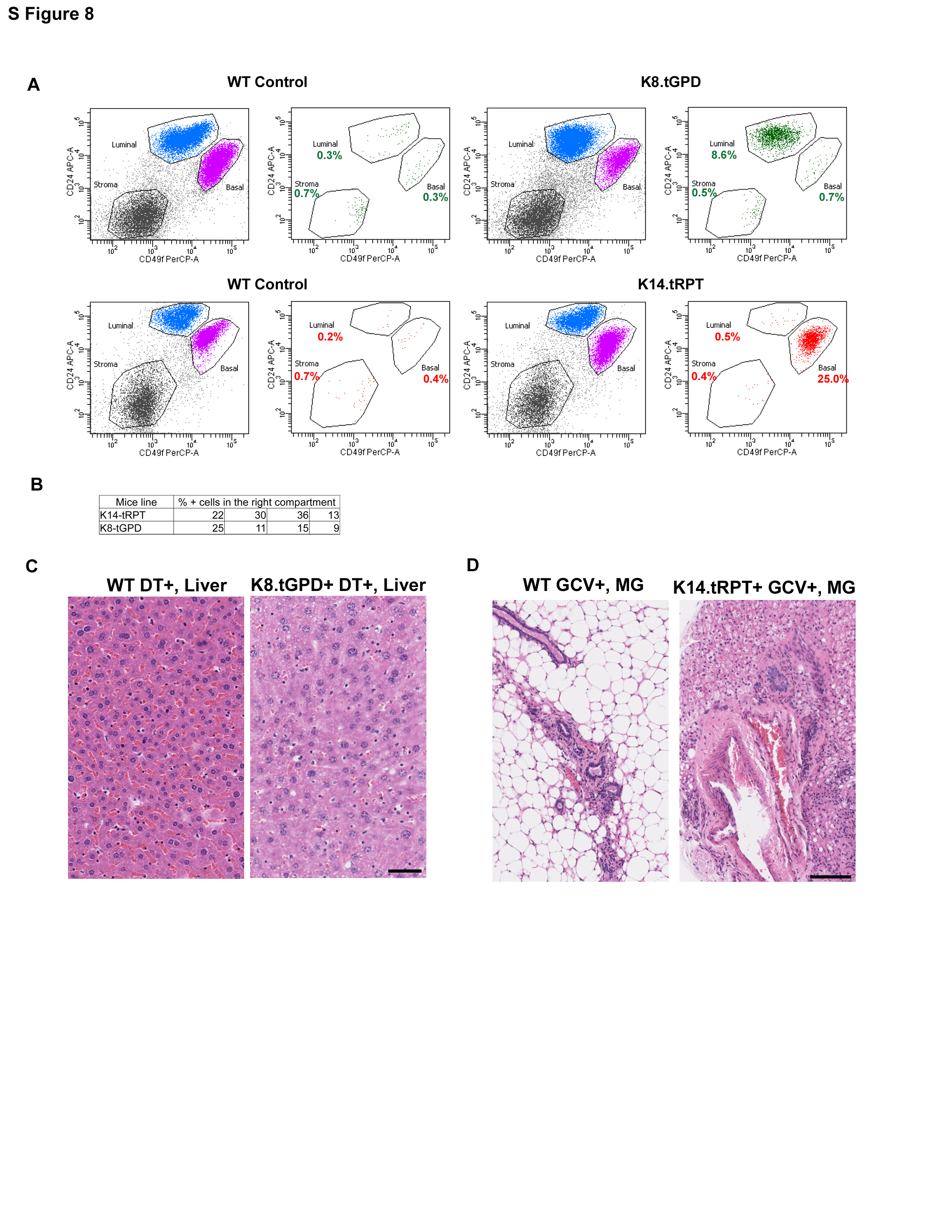

Supplement: S8 Fig — (A) Cells from mammary glands for either WT, K8.tGPD, or K14.tRPT mouse were analyzed by flow cytometry, and percentage of reporter-positive cells for stroma, basal, and luminal compartments are shown. The first dot plot shows the total population per compartment, whereas the second shows only the cells that are positive for the reporters. Gates were set based on the negative control, and percentages are given in a color-code manner for the reporter-positive cells. (B) Table summarizing results of A for multiple mice. Percentage of reporter-positive cells in the right compartment. (C) H&E images of liver from either control (WT) or K8.tGPD mouse after exposure to high-dose DT. Scale bar 50μm. (D) H&E images of MG of either control (WT) or K14.tRPT mouse after exposure to low-dose GCV. Scale bar 100 μm. GCV, ganciclovir; H&E, hematoxylin and eosin; K8.tGPD, keratin-8 promoter followed by turbo green fluorescent protein and diphtheria toxin receptor; K14.tRPT, keratin-14 promoter followed by a turbo red fluorescent protein and herpes simplex virus thymidine kinase; MG, mammary gland; WT, wild-type. (TIF) [file pbio.2004049.s008.tif]

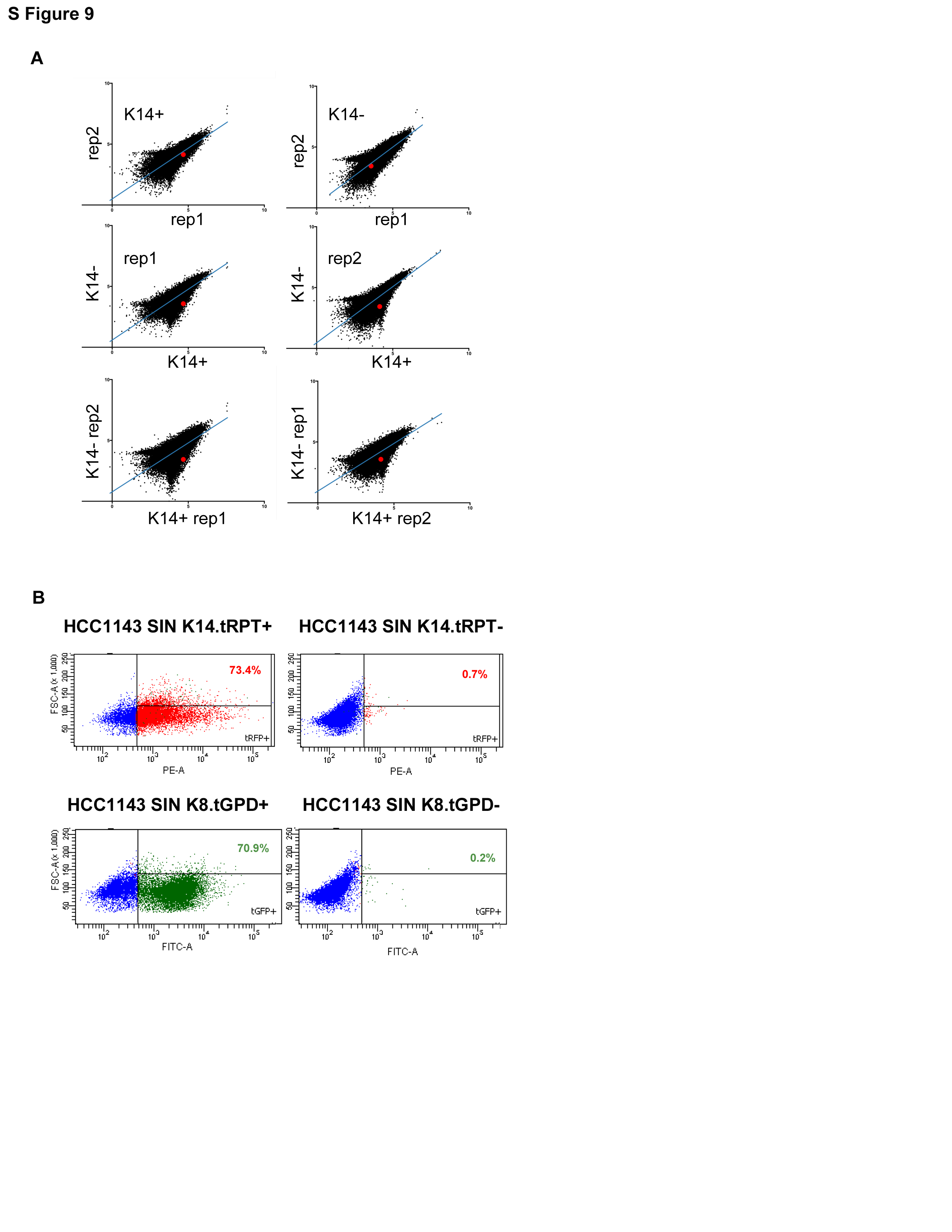

Supplement: S9 Fig — (A) Shows the scatter plots in which each dot is a peak of H3K27ac plotted according to the ChIPseq signal in the specified replicate (“rep”) of the indicated cell line. The red dots correspond to the peak at the Amigo2 promoter. (B) HCC1143 human breast cancer cell line expressing the SIN lentiviral K14.tRPT or K8.tGPD reporter were sorted for the constitutive expressed tBFP and subsequently sorted for tRFP (upper panels) or tGFP (lower panel). Amigo2, amphoterin-induced protein 2; ChIPseq, chromatin immunoprecipitation sequencing; H3K27ac, histone 3 lysine 27; K8.tGPD, keratin-8 promoter followed by turbo green fluorescent protein and diphtheria toxin receptor; K14.tRPT, keratin-14 promoter followed by a turbo red fluorescent protein and herpes simplex virus thymidine kinase; SIN, self-inactivating; tBFP, blue fluorescent protein; tRFP, turbo red fluorescent protein. (TIF) [file pbio.2004049.s009.tif]

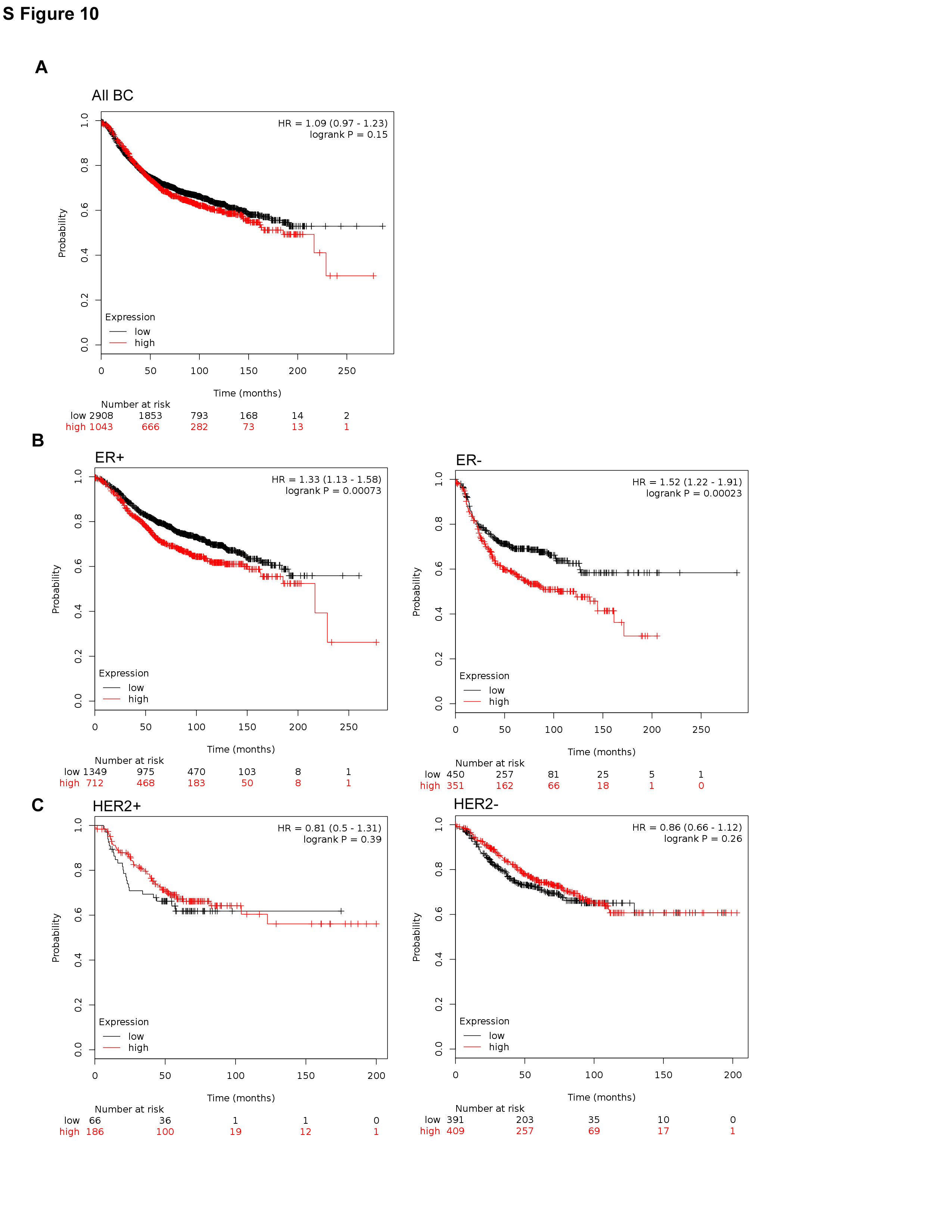

Supplement: S10 Fig — Kaplan-Meier plot in all (A) breast cancer (“all BC”), (B) ER− and ER+, and (C) HER2 amplification positive (HER2+) and negative (HER2−) breast cancer shows relationship between Amigo2 expression and relapse-free survival. Amigo2, amphoterin-induced protein 2; ER, estrogen receptor; HER2 human epidermal growth factor receptor 2. (TIF) [file pbio.2004049.s010.tif]

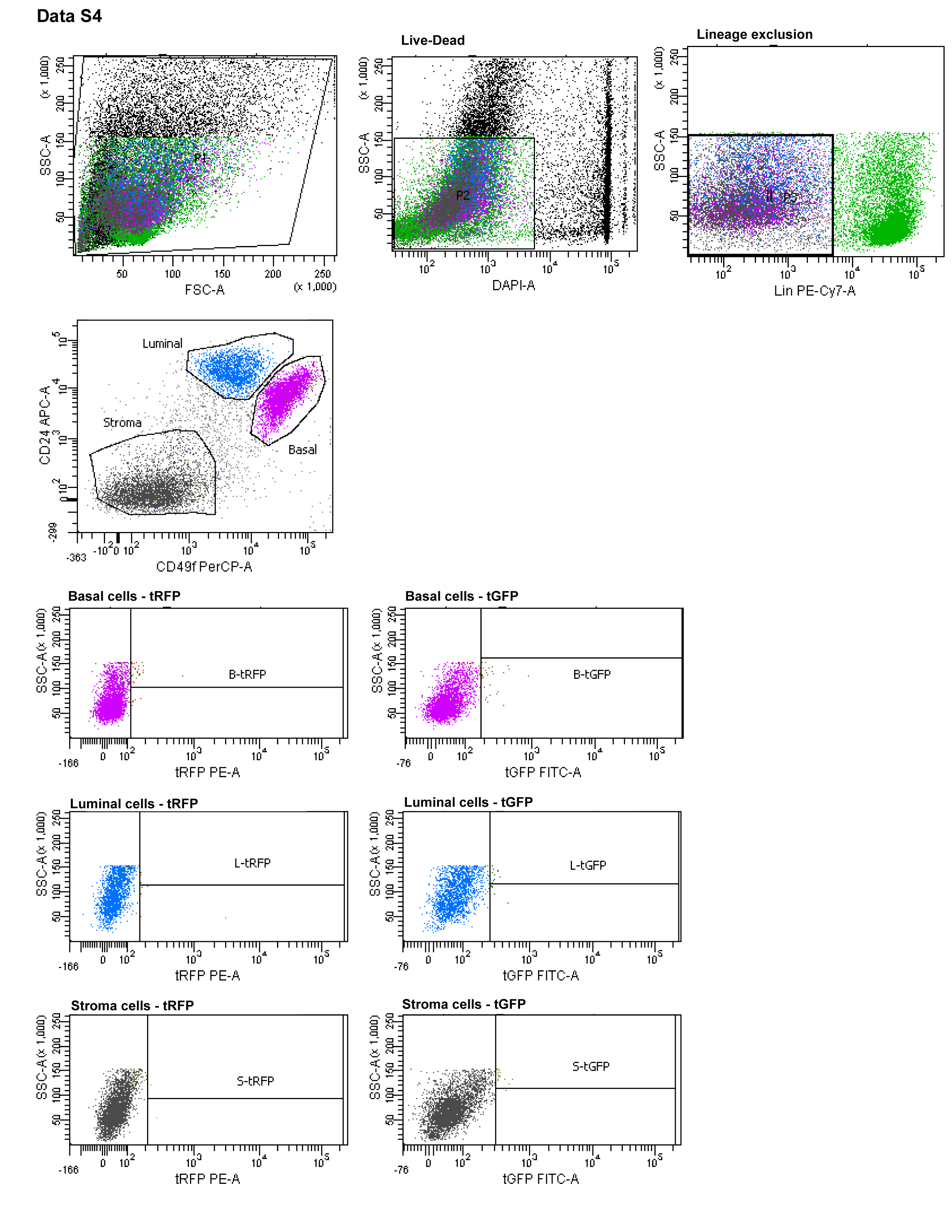

Supplement: S4 Data — (TIF) [file pbio.2004049.s014.tif]

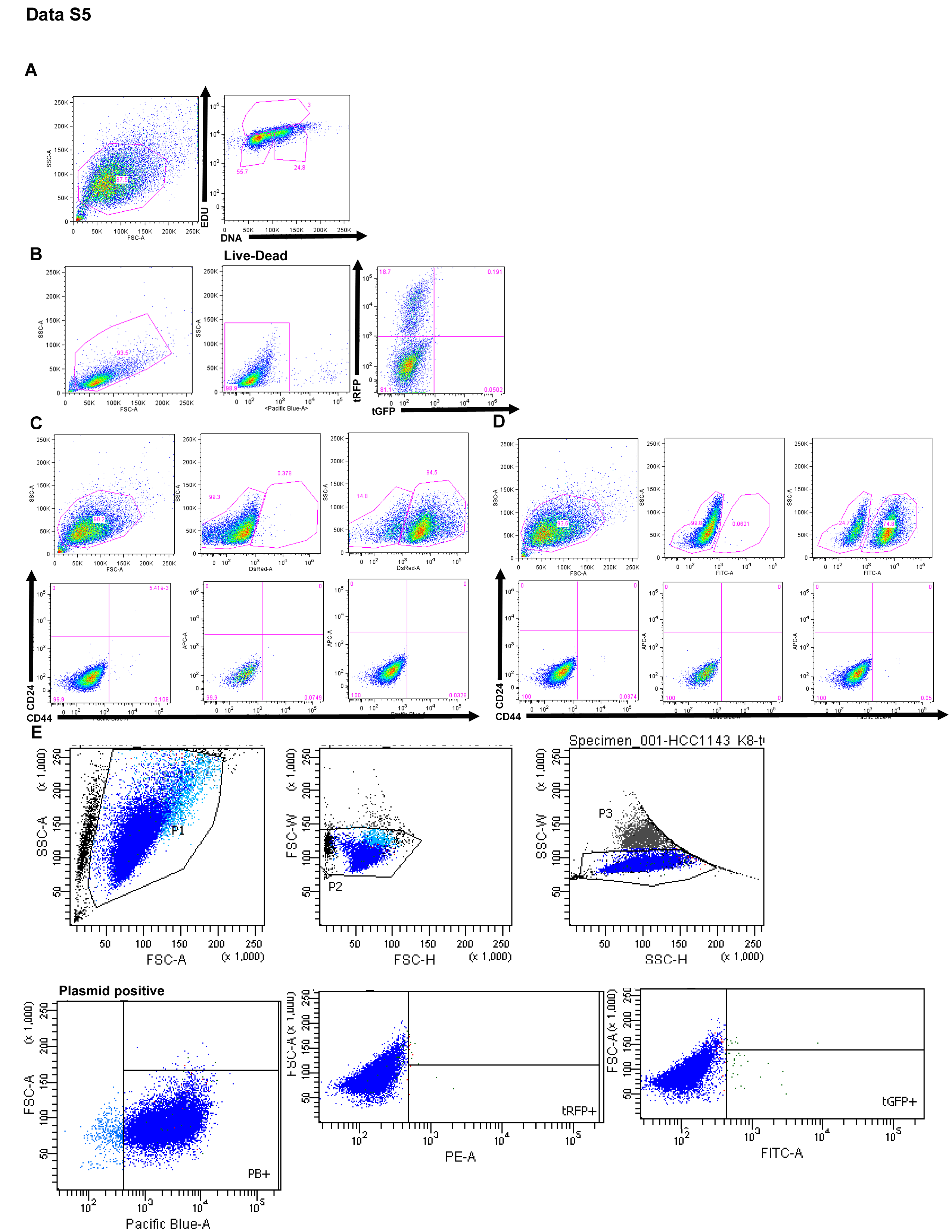

Supplement: S5 Data — Representative dot plot showing the gating strategy for (A) S1C and S5A Figs, (B) S5C Fig, (C) S7C Fig, (D) S7D Fig, and (E) S9B Fig. (TIF) [file pbio.2004049.s015.tif]
